# Circadian rhythmicity and biopsychosocial characteristics influence opioid use in chronic low back pain

**DOI:** 10.1172/JCI188620

**Published:** 2025-10-01

**Authors:** Doriana Taccardi, Amanda M. Zacharias, Hailey G.M. Gowdy, Mitra Knezic, Marc Parisien, Etienne J. Bisson, Zhi Yi Fang, Sara A. Stickley, Elizabeth Brown, Daenis Camiré, Rosemary Wilson, Lesley N. Singer, Jennifer Daly-Cyr, Manon Choinière, Zihang Lu, M. Gabrielle Pagé, Luda Diatchenko, Qingling Duan, Nader Ghasemlou

**Affiliations:** 1 Department of Biomedical and Molecular Sciences, Queen’s University, Kingston, Ontario, Canada.; 2 Faculty of Dental Medicine and Oral Health Sciences, Department of Anesthesia, Faculty of Medicine, Alan Edwards Centre for Research on Pain, McGill University, Montreal, Quebec, Canada.; 3 Kingston Health Sciences Centre, Kingston, Ontario, Canada.; 4 Department of Anesthesiology and Perioperative Medicine,; 5 Centre for Neuroscience Studies, and; 6 School of Nursing, Queen’s University, Kingston, Ontario, Canada.; 7 Chronic Pain Network, McMaster University, Hamilton, Ontario, Canada.; 8 Department of Anesthesiology and Pain Medicine, Université de Montréal, Montreal, Quebec, Canada.; 9 Research Center of the Centre hospitalier de l’Université de Montréal, Montreal, Quebec, Canada.; 10 Department of Public Health Sciences, Queen’s University, Kingston, Ontario, Canada.; 11 Department of Psychology, Université de Montréal, Montreal, Quebec, Canada.; 12 School of Computing, Queen’s University, Kingston, Ontario, Canada.

**Keywords:** Clinical Research, Inflammation, Neuroscience, Bioinformatics, Neutrophils, Pain

## Abstract

**BACKGROUND:**

Inter- and intraindividual fluctuations in pain intensity pose a major challenge to treatment efficacy, with a majority of people perceiving their pain relief as inadequate. Recent preclinical studies have identified circadian rhythmicity as a potential contributor to these fluctuations and a therapeutic target.

**METHODS:**

We therefore sought to determine the impact of circadian rhythms in people with chronic low back pain (CLBP) through a detailed characterization, including questionnaires to evaluate biopsychosocial characteristics, ecological momentary assessment (7 day e-diaries at 8:00/14:00/20:00) to observe pain fluctuations, and intraday blood transcriptomics (at 8:00/20:00) to identify genes/pathways of interest.

**RESULTS:**

While most individuals displayed constant or variable/mixed pain phenotypes, a distinct subset had daily fluctuations of increasing pain scores (>30% change in intensity over 12 hours in ≥4/7 days). This population had no opioid users, better biopsychosocial profiles, and differentially expressed transcripts relative to other pain phenotypes. The circadian-governed neutrophil degranulation pathway was particularly enriched among arrhythmic individuals; the link between neutrophil degranulation and opioid use was further confirmed in a separate CLBP cohort.

**CONCLUSION:**

Our findings identified pain rhythmicity and the circadian expression of neutrophil degranulation pathways as indicators of CLBP outcomes, which may help provide a personalized approach to phenotyping biopsychosocial characteristics and medication use. This highlights the need to better understand the impact of circadian rhythmicity across chronic pain conditions.

**FUNDING:**

This work was funded by grants from the Canadian Institutes of Health Research (CIHR; grant PJT-190170, to NG and MGP) and the CIHR-Strategy for Patient-Oriented Research Chronic Pain Network (grant SCA-145102, to NG, QD, LD, MGP, and MC). DT was funded by a MS Canada endMS Doctoral Research Award, AMZ by an Ontario Graduate Scholarship, HGMG by a CIHR Doctoral Research Award, MGP by a Junior 2 Research Scholarship from the Fonds de recherche du Québec – Santé, and LD by a Canadian Excellence Research Chairs and Pfizer Canada Professorship in Pain Research.

## Abstract 2

Rhythms in pain intensity and immune cell profiles are indicators of reduced opioid use and improved biopsychosocial characteristics, while arrhythmic pain profiles reflect poor outcomes.

## Graphical abstract

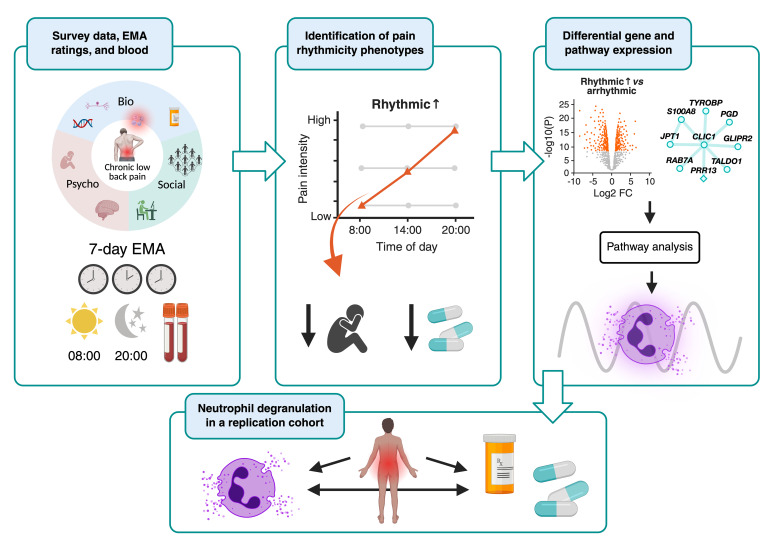

## Introduction

Chronic low back pain (CLBP), lasting more than 3 months, is the leading cause of years lived with disability worldwide and has increased significantly over the last 25 years, a trend likely to persist with the aging population (1, 2). This disease substantially impairs quality of life, resulting in a higher risk of anxiety and depression as well as reduced work and wages, creating a dependence on prescribed opioids used to treat the pain (3, 4). Comorbid factors, including lifestyle, occupation, and psychosocial variables, affect the prevalence and outcomes of CLBP, resulting in few effective management strategies (5, 6). Heterogeneity exists in the response to most therapeutic strategies, with only a subset of individuals benefiting from specific approaches (7, 8). Rather than treating pain with a one-size-fits-all solution, inter- and intraindividual variability in pain experiences must be considered, particularly with the advent of personalized and precision medicine (9, 10).

The standard of care for CLBP includes the use of NSAIDs, often in conjunction with muscle relaxants and tricyclic antidepressants (5, 11); opioids are also regularly prescribed even though their use for CLBP is not recommended (12, 13). Long-term use of opioids results in higher prescribed doses, longer-acting preparations, and titration with open-ended dose escalation (14). This leads to an increased risk of deleterious side effects (e.g., drowsiness, constipation, tolerance, hyperalgesia, physical dependence, and withdrawal) (15) and has contributed to the global epidemic of opioid misuse (16, 17). Recent evidence now points to a detrimental effect of NSAIDs in the transition from acute to CLBP by altering the neutrophil response (18). Retrospective analysis of patient data now finds peri-operative NSAID use to be associated with increased odds of continued opioid prescription postoperatively in knee arthroplasty (19), which could also have ramifications for the treatment of CLBP. These drugs act to reduce inflammation by inhibiting cyclooxygenases that synthesize prostanoids, which are primarily expressed by myeloid cells such as neutrophils and macrophages (20). Regulated activity of these immune cells, particularly in the acute stage, is necessary for the generation of pain and subsequent resolution of inflammation (21).

The inflammatory response to injury is tightly orchestrated, with distinct neuroimmune interactions contributing to both acute and chronic pain (22). An added measure of complexity that has not been widely considered in the context of pain is the rhythmic nature of immune cells. Circadian (24 hour) rhythms, present in most cells, are composed of tightly regulated clock genes that control transcriptional–translational feedback loops (23). These genes have been shown to alter expression of inflammatory mediators that may play a role in the generation and maintenance of pain (24–26).

Evidence shows a reciprocal relationship between disrupted circadian rhythms and altered pain thresholds, suggesting the existence of a feedback loop between the experience of pain and the circadian system (26). This response is not only restricted to peripheral cells, but may also include central glia (e.g., astrocytes, microglia, and oligodendrocytes) that contribute to the chronic pain response. Furthermore, the sensory neurons that lie at the interface of the peripheral and central nervous systems could also contribute to altered immunity (27) via rhythmic expression of inflammatory mediators (28–32). Thus, understanding changes in circadian rhythmicity (such as in immune cells and neurons) becomes important in the context of pain and other chronic diseases.

Rhythmicity in innate immune cells, particularly neutrophils and monocytes, has been well characterized in preclinical models as well as in healthy and diseased states in clinical populations (33). In neutrophils, these rhythms drive their efflux from the bone marrow and into the bloodstream, with chemotactic signals then recruiting these cells into specific tissues and organs (34–36). Like neutrophil rhythmicity, pain is also variable and dynamic, both within the day and between individuals with the same disease (25). The effect of these rhythms on chronic pain intensity, immune response, and biopsychosocial variables remains unexplored despite evidence that circadian rhythms regulate the nervous and immune systems and that misalignment of these clocks impacts pain and other neuroinflammatory diseases (37, 38).

The causes or implications of pain fluctuation remain unclear, and no specific biomarkers have been identified to track rhythmic changes in chronic pain. Therefore, we sought to investigate whether variability in pain intensity could be linked with biopsychosocial profiles and immune cell signatures. We believe that our study provides a proof-of-concept that individual rhythmicity of pain intensity and neutrophil activation may serve as tools to stratify patients with CLBP using a biopsychosocial approach; surprisingly, we found that these individual differences may serve as a guide to opioid prescription. This work represents a step toward using intraday changes at the molecular, cellular, and system levels as a precision medicine approach to understand and treat chronic pain.

## Results

### Pain rhythmicity as a tool to phenotype CLBP

**Figure 1:**
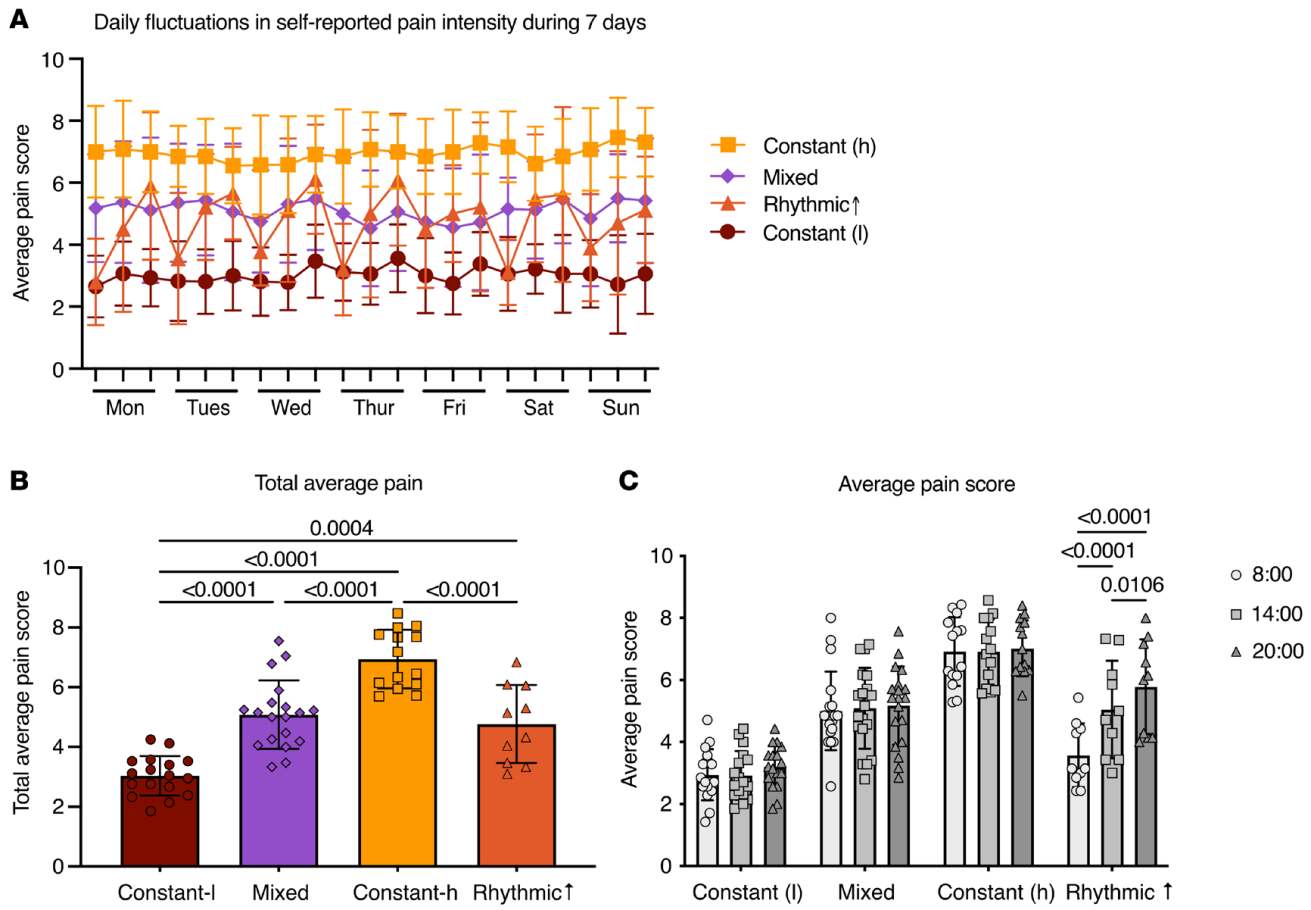
CLBP cohort grouped by pain intensity on a 24 hour scale (*n* = 60). (**A**) Pain intensity was recorded at 8:00, 14:00, and 20:00 over a 7 day sampling period with an EMA e-diary tool. Average pain intensity reported by CLBP participants is classified as following 1 of 4 distinct patterns: consistent pain across all time points with a low mean (constant-low), consistent pain with a high mean (constant-high), an unpredictable phenotype (mixed), and rhythmic pain with a peak in intensity occurring in the evening (rhythmic↑). (**B**) Total average score in pain intensity among groups. Individuals belonging to the constant-high group reported the greatest mean pain intensity relative to all other pain clusters. (**C**) Average score in pain intensity among groups. A collective average for the 7 day sampling period was calculated for each participant at each time point (8:00, 14:00, and 20:00). A significant change in pain scores was identified in the rhythmic↑ group with an upward trajectory throughout the day. All data were analyzed using an ordinary 1-way ANOVA with post hoc Tukey’s test and are represented as mean ± SD.

A total of 74 patients participated in the study, with 12 excluded for either incomplete electronic diary (e-diary) data or for no longer meeting the eligibility criteria; 62 participants were included for final analysis (Table 1 and Supplemental Figures 1 and 2; supplemental material available online with this article; https://doi.org/10.1172/JCI188620DS1). Of these, 35 participants identified as female and 27 as male, with a mean age of 51.8 years (SD 14.4); 91.9% identified as White. Ecological momentary assessment (EMA) data were collected at 8:00, 14:00, and 20:00 daily over 7 days, similar to time points used in other studies (39, 40), and were used to determine low/high variability groups divided by the 50th percentile of daily pain score SD. We began by identifying patients with low variability in pain scores, who were further divided based on mean pain intensity at the 50th percentile, resulting in 2 populations: constant-low (*n* = 17) and constant-high pain (*n* = 14) (Figure 1A). Importantly, these patients did not exhibit a clinically significant (≥30%) change in average self-reported pain scores between 8:00 and 20:00 on most days. Patients with variable pain scores were grouped using the directionality and magnitude of daily change in pain intensity, again using the 30% change in pain intensity as a threshold (Supplemental Figures 3 and 4). Those with more intense pain at night on the majority of days (≥4 of 7 days) were identified as rhythmic increasing (rhythmic↑; *n* = 10); only 2 participants exhibited a rhythmic decreasing phenotype (rhythmic↓; Supplemental Figures 1 and 2) and thus were excluded from further analysis. The remaining patients (*n* = 19) had unpredictable or mixed pain patterns. Only the rhythmic↑ phenotype had an aggregate pain intensity score similar to the mixed population (Figure 1B) and exhibited significant differences in pain scores over the 3 times of day assessed (*P* < 0.0106; Figure 1C).

### Pain rhythmicity is associated with improved psychosocial profiles

**Figure 2:**
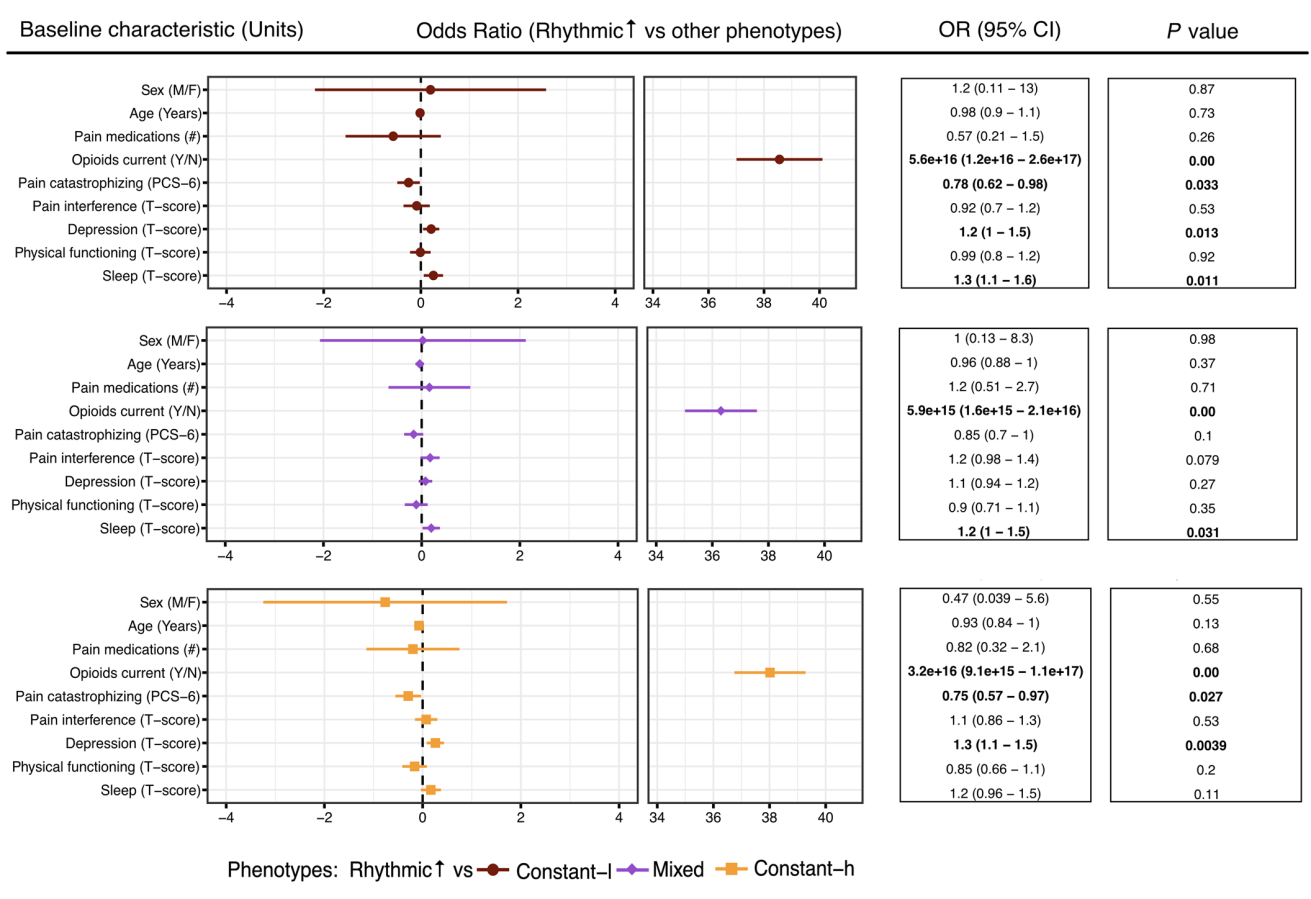
Biopsychosocial measures from the CLBP cohort differ across grouped pain phenotypes (*n* = 60). Multinomial logistic regression of psychosocial variables. Numbers of pain and opioid medications were included in the medical history questions. Sleep disturbance, pain interference, physical functioning, and depression were measured using PROMIS-29 v2.0 scales; results are presented as *t*-scores, where 50 is indicative of the score in an average general American population. A 10 point difference in *t*-score represents a change of 1 SD difference in the severity of the assessed behavioral factors. Pain catastrophizing was measured with PCS-6, range 0–24; a higher score indicates a greater degree of pain catastrophizing. Plotted in the *x* axes are the regression coefficients β (log odds). ORs are reported with 95% CIs in parentheses. *P* values are shown with significant results in bold (*P* < 0.05).

While the constant-low and -high pain groups exhibited significantly different average intensity scores compared with all other groups (Figure 1B), we focused our subsequent analyses on understanding the impact of pain rhythmicity on biopsychosocial profiles. The rhythmic↑ phenotype showed improved biopsychosocial profiles compared with the 3 other phenotypes, with higher ORs for depression in the constant-low and -high phenotypes and sleep disturbance in the constant-low and mixed phenotypes (Figure 2). Increased ORs of 1.2 (95% CI 1–1.5, *P* = 0.013) and 1.3 (95% CI 1.1–1.5, *P* = 0.004) were observed for depression in the constant-low and -high groups, relative to the rhythmic↑ phenotype, respectively. There was also increased OR of sleep disturbance in the constant-low and mixed pain phenotypes of 1.3 (95% CI 1.1–1.6, *P* = 0.01) and 1.2 (95% CI 1–1.5, *P* = 0.03). Surprisingly, reduced OR for pain catastrophizing was identified in the constant-low (0.78 [95% CI 0.62–0.98, *P* = 0.033]) and constant-high groups (0.75 [95% CI 0.57–0.97, *P* = 0.027]) relative to the rhythmic↑ group. A separate multinomial logistic regression, including sleep apnea, psychological disorder, and psychological counseling as covariates, was completed. The ORs for having received psychological counseling were not significant (*P* > 0.12) across pain phenotypes, whereas ORs for sleep apnea and psychological disorder were significant (*P* < 0.001; Supplemental Figure 5).

Next, we used the same pain intensity variability and mean score stratification strategy (described above) to assess whether EMA data collected for fatigue and mood could also be used to group patients as we did for pain. We found that while similar populations exist using mood and fatigue scores (Supplemental Figure 3), the data obtained via EMA were not suitable for phenotyping patients. This is likely because the population recruited were identified as reporting chronic pain as their primary condition, rather than for fatigue or depressive mood. Despite this, we found that mean daily scores for pain were significantly correlated with mood (Spearman’s correlation: *r*_s_ = 0.479, *P* < 0.0001) and fatigue (*r*_s_ = 0.659, *P* < 0.0001), with mood and fatigue also significantly correlated with each other (*r*_s_ = 0.672, *P* < 0.0001) (Supplemental Figure 4). Thus, from our main regression analysis, we concluded that pain rhythmicity, but not that of mood or fatigue, is associated with improved psychosocial profiles (Figure 2).

Though each phenotype differed greatly in terms of psychosocial characteristics, there were no significant differences regarding the sociodemographic factors of the individuals in these groups, including sex and gender distribution, age, education levels, household incomes, BMI, and smoking prevalence (categorical variables: Fisher’s exact tests; continuous variables: Kruskal-Wallis tests; Table 1). Furthermore, no significant differences were observed in the time from onset of low back pain (LBP) between phenotypes. There were few significant differences in the LBP-related medical history of participants when comparing the prevalence of back pain history factors; individuals with constant-high or mixed phenotypes were more likely to report LBP every day or nearly every day at the time of assessment (Fisher’s exact test, *P* = 0.041; Supplemental Table 1) than all others. Additionally, individuals with these phenotypes reported “being more bothered” by pain in the shoulder, neck, or upper back (*P* = 0.006) and widespread pain (*P* = 0.014) than individuals with constant-low or rhythmic↑ phenotypes (Supplemental Table 1). There were no significant differences when comparing the reception of LB operation, injection treatment, or exercise therapy among phenotypes (Supplemental Table 1) nor were there significant differences between the prevalence of comorbidities (Supplemental Table 2).

### Pain rhythmicity is associated with reduced opioid use and antidepressants

Opioid use was a common and highly significant factor when comparing the rhythmic↑ phenotype with all others (multinomial logistic regression [MLR] OR ≥ 3.2e+16, *P* < 0.0001, Figure 2; Fisher’s exact test: *P* = 0.008, Table 2). Indeed, none of the rhythmic↑ patients were prescribed an opioid to treat their pain, relying instead on NSAIDs, antidepressants, and other medications (Table 2). The mixed and constant-high phenotypes were prescribed more medications to treat their pain than the constant-low and rhythmic↑ groups, even while the mixed and rhythmic↑ phenotypes had similar average pain scores. Antidepressant use showed a similar trend among the groups, with the rhythmic↑ phenotype using fewer medications relative to other groups (*P* = 0.016; Table 2). Indeed, only 1 participant of the 10 in the rhythmic↑ group reported the usage of antidepressants to treat their pain, which was comparable with 2 of 17 participants (11.8%) in the constant-low group, while almost half of the people in the mixed (42.1%) and constant-high (57.1%) groups used antidepressants for their pain.

### Immune cell activation is associated with pain rhythmicity

**Figure 3:**
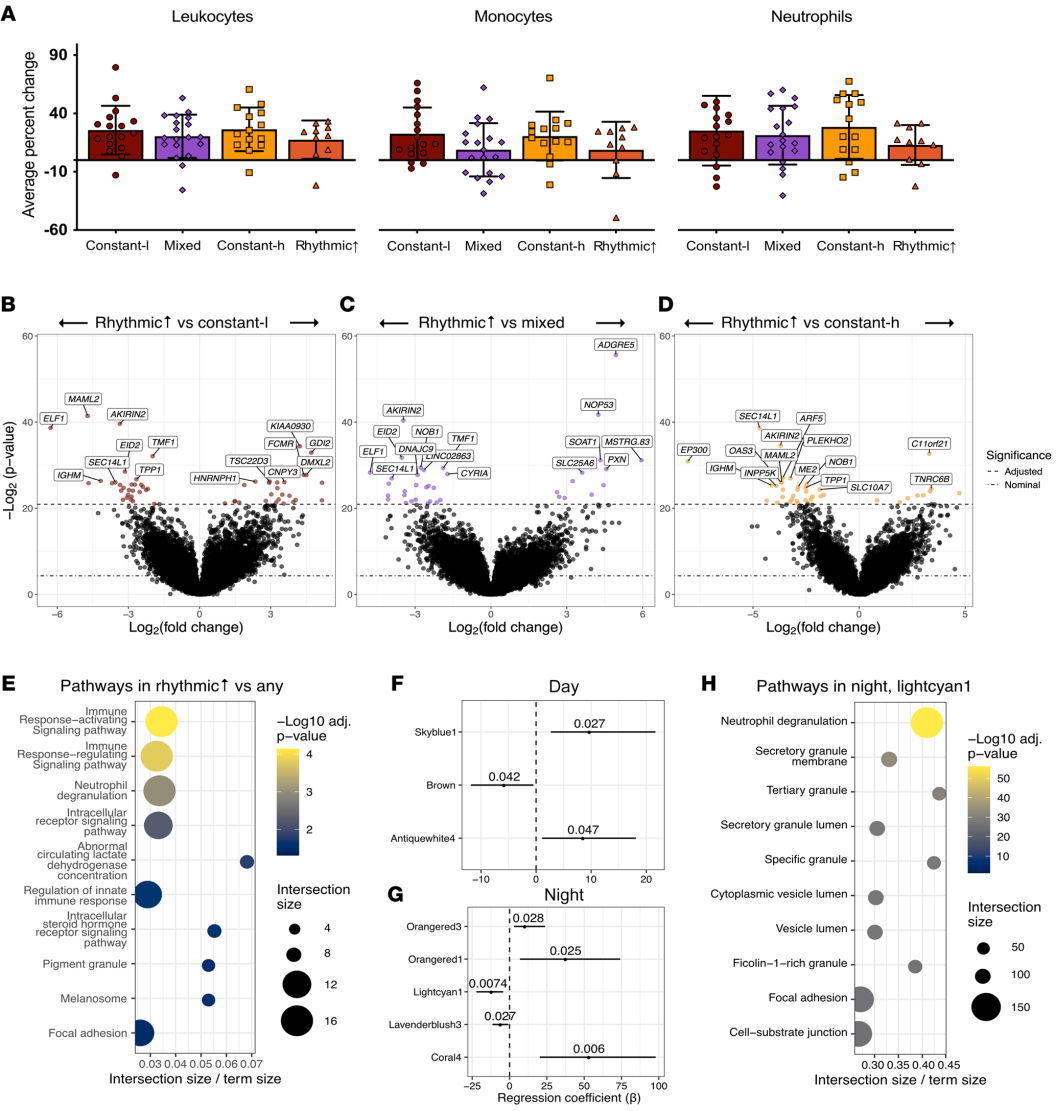
Complete blood cell count (*n* = 120) and transcriptomic analysis in the CLBP cohort highlight neutrophil activation as important to pain rhythmicity (*n* = 116). (**A**) Complete blood cell count with differential was performed on peripheral blood (leukocytes, monocytes, and neutrophils) collected at 8:00 and 20:00, and the percentage change in cell quantity was calculated. No significant differences were observed in percentage change of leukocytes, monocytes, or neutrophils. Data were analyzed using a 1-way ANOVA with post hoc Tukey’s test. Data are represented as mean ± SD. (**B**–**D**) Volcano plots of DETs (colored points: constant-low, *n* = 55; mixed, *n* = 37; constant-high, *n* = 39) between the rhythmic↑ phenotype and other phenotypes (*P*_Bonferroni_ < 0.05). The top 15 significant transcripts are labeled by their respective genes. (**E**) Pathway enrichment analysis of genes corresponding to DETs between the rhythmic phenotype and any other phenotype. The top 10 significant pathways (*P*_g:SCS_ < 0.05, pathway size ≥ 10 and ≤ 500) are shown on the *y* axis. (**F**–**G**) The association between transcript coexpression clusters and the rhythmic↑ phenotype was assessed using a GLM. Forest plots represent clusters significantly correlated with phenotype in the day network (top; *P* < 0.05) and the night network (bottom; *P* < 0.05). The *x* axes are the regression coefficient β (relative log odds). *P* values are reported as numbers. (**H**) Pathway enrichment analysis of the night, lightcyan1 cluster’s respective genes. The top 10 significant pathways (*P*_g:SCS_ < 0.05, pathway size ≥ 10 and ≤ 500) are shown on the *y* axis.

Given that immune cells are key players in endogenous peripheral opioid production, we sought to determine whether pain rhythmicity depends on the immune response by analyzing whole blood collected at 8:00 and 20:00. Immune cell numbers and percentage change over 12 hours were unchanged across the pain rhythmicity groups (Figure 3A and Supplemental Figures 6 and 7). Additionally, quantitative PCR (qPCR) on peripheral blood did not show any significant differences in clock gene expression in the morning versus evening, except for *PER1*,which presented a higher level of expression at 8:00 relative to 20:00 in both the constant-low and rhythmic↑ pain phenotypes (Supplemental Figure 8); these results are similar to those in healthy controls (41). We used RNA sequencing of whole blood to determine whether there were any changes in activation state. This analysis yielded 37–169 differentially expressed transcripts (DETs) between the rhythmic↑ group and any other pain phenotypes (*P*_Bonferroni_ < 0.05; Figure 3, B–D), the majority of which (12–144 DETs) were downregulated in the rhythmic↑ phenotype relative to the other groups. Pathways enriched among these DETs implicated immune cell signaling mechanisms, with neutrophil degranulation specifically enriched (*P*_g:SCS_ < 0.05; Figure 3E). Furthermore, candidate differential expression analysis of neutrophil-related inflammatory response pathways revealed that transcripts corresponding to the S100A8 gene had higher expression among constant-low phenotype participants (*P*_Bonferroni_ < 0.05; Supplemental Figures 9 and 10).

Gene coexpression network analysis, which identifies highly correlated transcripts (grouped by module), found 82 modules of coexpressed transcripts at 8:00 and 77 modules at 20:00; of these, 8 modules were specifically associated with the rhythmic↑ phenotype (generalized linear model [GLM] *P* < 0.05; Figure 3, F and G). The neutrophil degranulation pathway was highly enriched in a module negatively associated with the rhythmic↑ phenotype, both as a binary and multinominal factor (Figure 3H; *P*_g:SCS_ = 9.51E^–57^). Since network modules are often used as therapeutic targets or candidate biomarkers, neutrophil degranulation may be important in differentiating the rhythmic↑ phenotype from others.

Given the correlation between opioid consumption and pain rhythmicity, we sought to determine whether there was differential gene expression between opioid users and nonusers when omitting the rhythmic↑ patients from the analysis. We found only 17 transcripts were differentially expressed (*P*_Bonferroni_ < 0.05; Supplemental Figure 11, A and B), and the neutrophil degranulation pathway was not enriched among the constant/mixed phenotypes, further strengthening the link between pain rhythmicity, opioid use, and neutrophil activation.

While we found equivalent use of pain medications across the 4 pain phenotypes, there were significant differences in antidepressant, opioid, and pain medication polypharmacy among our patients (Table 2). To understand whether the rhythmicity effect found in our cohort was specific to opioid use, we stratified nonrhythmic participants into opioid and nonopioid users, as in our transcriptomic analyses. We found no changes in pain score at 8:00, 14:00, and 20:00 in this analysis (Supplemental Figure 11C).

### Neutrophil degranulation depends on opioid use in a replication cohort with chronic pain

**Figure 4:**
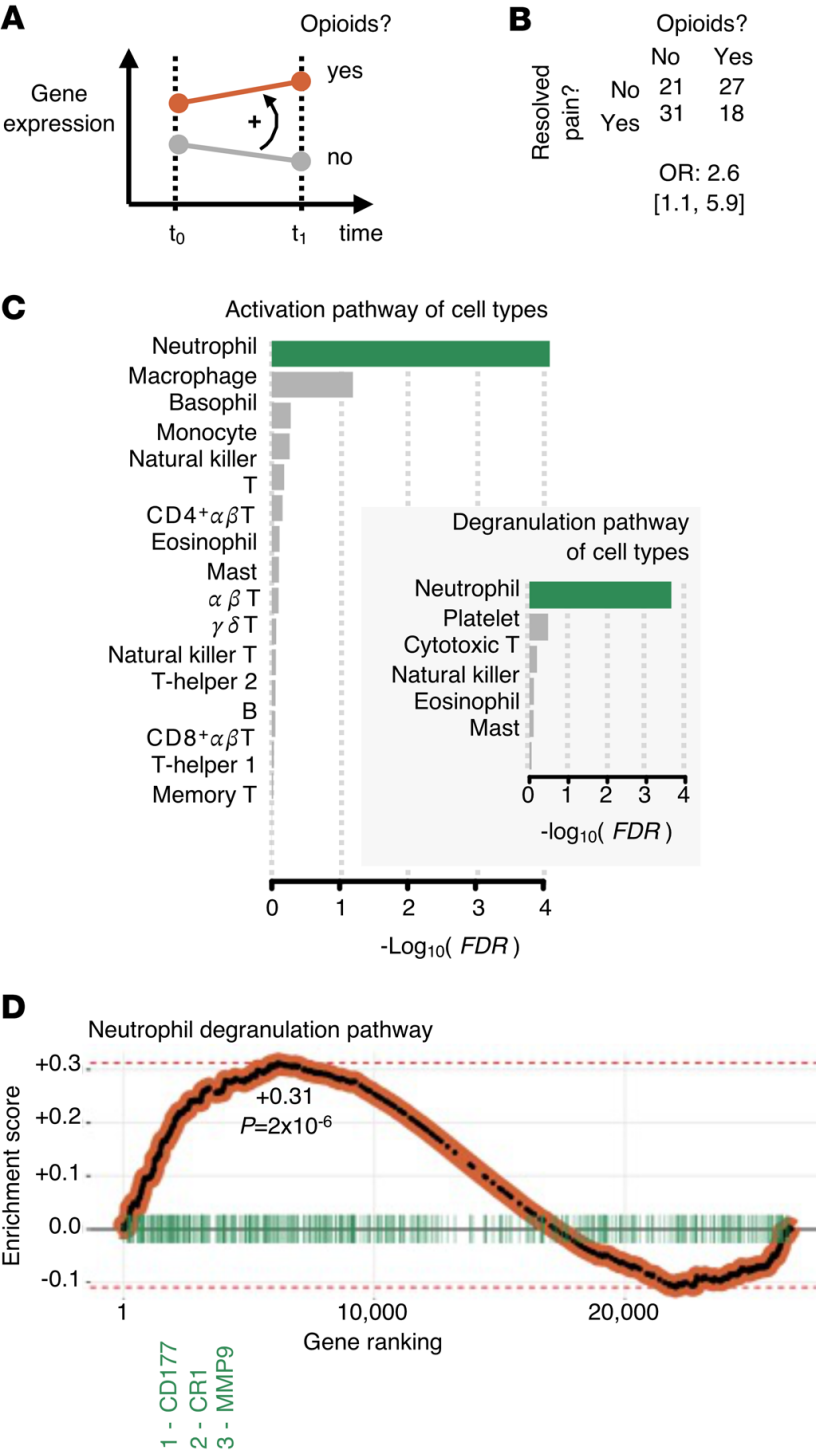
Previous findings were replicated in an independent transcriptomic LBP cohort. (**A**) Transcriptomics study design. Whole-blood transcriptomics were determined at a baseline visit (*t*_0_) and at a 3 month follow-up visit (*t*_1_), enabling tracking of gene expression with time in those using opioids and in those who were not. Regression of gene expression on an opioid use × time interaction term determines if the time trajectory of expression of a gene in opioid users (orange) differs from those not using (gray). The sign of the effect of the interaction term will illustrates how the time trajectory of nonusers must be rotated to match that of users, in this case, counterclockwise when positive (black curved arrow). (**B**) Number of opioid users versus number of participants whose acute back pain resolved by the time of the follow-up visit. OR and 95% CI are indicated for pain persistence versus opioid intake. (**C**) Enrichment of activation pathways across cell types. Enrichment for cell degranulation pathways across cell types of hemopoietic lineage. Bars in green indicate FDR < 1%. (**D**) Pathway enrichment plot for the neutrophil degranulation pathway. All genes were ranked from the most positive effect of opioid use × time (left) to the most negative (right). Genes in the neutrophil degranulation pathway are shown (vertical green bars), along with the 3 top-ranking genes (far left). The pathway’s enrichment score and unadjusted *P* value are indicated.

Finally, we sought to confirm whether pain chronicity was associated with opioid use and immune cell activity. Using an independent cohort of patients presenting with LBP and assessed again 3 months later (*n* = 97), we asked if gene expression in whole blood cells differed over time. We grouped participants in the replication cohort by opioid use (3 months after onset of LBP; *n* = 45 opioid users and 52 nonusers) using the absence of opioid use as a proxy for pain rhythmicity (Figure 4A). A census of pain outcomes at the study follow-up visit indicated significantly increased odds for pain chronicity among those consuming opioids (OR = +2.6, 95% CI 1.1−5.9) (Figure 4B), indicating that gene expression assessment of opioid consumption must be corrected for pain phenotypes.

Differential expression analyses over 3 months found no genes whose expression was significantly different with time between opioid users and nonusers (FDR > 0.05; Figure 4, A and B). Only the neutrophil activation pathway was enriched among all activation pathways by hematopoietic cells (FDR < 1%; Figure 4C). Furthermore, neutrophil degranulation was also enriched among all pathways of blood cell types capable of degranulation (Figure 4C). The neutrophil degranulation pathway enrichment score indicated that the genes in those using opioids were overexpressed with time compared with nonusers (Figure 4D, left side of plot; +0.31, *P* = 2 × 10^–6^). Time trajectories of gene expression levels in opioid users and nonusers were confirmed for the top 3 pathway genes (*CD177*, *CR1*, and *MMP9*) and indicated downregulation of expression among opioid users to a nominally significantly (*P* ≤ 0.05) lesser degree than nonusers (Supplemental Figure 12). Thus, our results suggest that neutrophil degranulation is associated with pain rhythmicity on a 24 hour scale (CLBP main cohort) and opioid use in LBP (replication cohort).

## Discussion

This observational study assessed whether and how pain rhythmicity is associated with biopsychosocial profiles and immune cell activation in a population with CLBP. We found that biopsychosocial profiles were linked with pain rhythmicity by grouping patients into 4 distinct phenotypes. Those with constant-low or rhythmic↑ pain scores over 12 hours used fewer opioids, antidepressants, and other pain medications compared with those with mixed and constant-high phenotypes. Notably, the rhythmic↑ group had a similar average pain intensity across all time points as the mixed phenotype and a comparable score at night to the constant-high phenotype. Analysis of the peripheral immune response pointed to neutrophil degranulation as key to pain rhythmicity and, in a separate patient cohort, to opioid use in LBP chronicity. Our work lays the foundation for a better understanding of how circadian rhythms and immune cell activation work together to control chronic pain and opioid use.

Overall, the rhythmic↑ phenotype was associated with improved biopsychosocial profiles, particularly when considering depression and sleep disturbance. The presence of sleep apnea in approximately 22% of the mixed and constant-low phenotypes may have contributed to higher sleep disturbance in these 2 groups; however, sleep apnea was not significant when comparing comorbidities among all phenotypes (see Supplemental Table 2). Interestingly, there were approximately 50% fewer participants in the mixed and rhythmic↑ groups who had received psychological counseling for LBP (see Supplemental Table 1 and Supplemental Figure 5). This may explain the reduced OR for pain catastrophizing in the constant pain phenotypes, as psychological counseling is known to mediate overall outcomes of chronic back pain treatment (42). Accordingly, people in the rhythmic↑ group reported a higher pain catastrophizing score (see Figure 2 and Supplemental Figure 5), which could put them at risk of experiencing a higher emotional burden of pain. Our work suggests that rhythmicity in CLBP may act as a potential indicator of biopsychosocial profiles, including pain catastrophizing and depression. Understanding health outcomes is likely incomplete without considering disease rhythms when designing clinical studies or assessing the efficacy of treatment strategies.

Measures of psychosocial factors are commonly recorded on a single occasion or represent an average retrospective score. However, EMA, facilitated by digital tools that allow for time-stamped data collection, is being used more often to record various symptoms, including pain intensity (9, 43). While only a few pain studies have tracked intraday changes (25, 44, 45), rhythmicity has not been thoroughly explored on an individual, molecular, and population level. Only recently, the interest in diverse pain rhythms has started to take hold: a comprehensive systematic review of circadian pain patterns in humans found that chronic pain intensity is highly variable, reaching higher scores either in the morning (e.g., for migraine, fibromyalgia, and osteoarthritis) or in the afternoon/evening (e.g., for tension headache, neuropathic pain, and temporomandibular joint pain) (44). Thus, considering the diverse etiologies of chronic pain and the variability in pain rhythmicity based on these different etiologies, it will be important to thoroughly characterize rhythmicity across diverse conditions of chronic pain. This is especially true for syndromes of mixed etiologies like CLBP, which can have nociplastic, neuropathic, and mixed components. However, studies have not explored a specific etiology of pain in depth, except for headache and migraine rhythmicity, which have been phenotyped with several studies including more than 1,500 participants (46–48).

While our main cohort included 74 people with CLBP and no other competing pain condition when recruited in the study at triage, some of these individuals reported some components of widespread pain in the medical history questionnaires. This was particularly observed among the mixed and constant-high groups (Supplemental Table 1). The complex, mixed etiologies present in CLBP and/or mechanisms underlying central sensitization may help explain this result (49). However, it is unclear if the phenomenon of central sensitization itself can cause chronic pain, especially in humans (50). Overall, our results reflect that CLBP is heterogeneous in terms of rhythmicity, regardless of underlying etiology. Future studies could address the contribution of central sensitization by integrating self-reported measures of pain across multiple times of day, alongside neurophysiological measures. This would help evaluate whether pain levels and rhythmicity are associated with a profile indicative of central sensitization (50). An examination of the underlying etiology of CLBP and its relation to pain rhythmicity would also be valuable.

While circadian rhythms and sleep are closely intertwined, with rest/wake cycles as an output variable in circadian studies, they represent different systems. Circadian rhythms can persist and are self-regulated in the absence of environmental cues by core clock genes including the positive (*BMAL1* and *CLOCK*) and negative (*PER1/2* and *CRY1/2*) components (23). Disruption of core circadian genes results in altered sleep, as do changes in external factors such as nutrition and activity (51–53). Thus, there is an interconnectedness that exists between sleep and circadian rhythmicity that is difficult to disentangle. While preclinical models suggest an association between sleep and pain sensitivity (54, 55), there is no evidence of this in healthy or clinical populations (56, 57). Therefore, further research is necessary to define the contribution of circadian rhythmicity at an individual cell level to pain outcomes and to understand how preclinical models of sleep disruption affect the circadian clock.

Circadian rhythms control most physiological processes and are especially important to the regulation of both the nervous and immune systems, which are key to pain chronicity. Less is known about the causal relationship between circadian rhythmicity and pain (24). A recent study showed that endogenous circadian rhythms, not sleep, accounted for most of the rhythmicity observed in thermal sensitivity among healthy subjects (57). This, in combination with the findings of the aforementioned studies in chronic pain populations (24, 25), suggests that circadian rhythmicity may contribute to chronic pain. The pain rhythmicity we observed likely depends on rhythmic neutrophil activation and degranulation. Key to our discovery is previous work showing that neutrophils (a) protect against the transition from acute to chronic LBP (18), (b) alleviate pain by releasing endogenous opioid peptides upon degranulation, and (c) are among the most rhythmic cells produced by the bone marrow (34). Patients with rhythmic↑ CLBP in our cohort showed a neutrophil signature associated with better overall biopsychosocial profiles than other groups. Thus, neutrophils may not only be important for predicting the transition to chronic pain (18), but also for detecting outcomes when chronic. Of note, the key genes identified in our replication cohort are also highly expressed among neutrophils (58).

Previous work suggested increased use of opioids in those with CLBP (12, 13). However, none of the rhythmic↑ patients in our cohort were using opioids, compared with 44% of the constant and mixed pain groups that did. A question that arises is whether opioid use disrupts circadian rhythmicity or if circadian gene expression influences opioid use in CLBP. There is evidence that opioids disrupt circadian rhythms in preclinical models (59–61), but this has been less well studied in clinical populations. Postmortem brain samples of people with opioid use disorder showed disrupted circadian rhythmicity in the nucleus accumbens and dorsolateral prefrontal cortex, key regions implicated in reward and opioid use (61, 62). Another study found disrupted rhythms of the circadian genes *PER1* and *PER2* in peripheral mononuclear cells collected from abstinent heroin users relative to healthy controls (63).

We show that intraday variability of pain intensity and gene expression among immune cells may represent key contributors to biopsychosocial characteristics in CLBP. It is possible that differences in DETs among the rhythmic↑ phenotype (versus other groups) may be due to opioid use and not differential neutrophil degranulation. If this were the case, we would expect neutrophil activation genes to be differentially expressed between opioid users and nonusers, even when excluding the rhythmic phenotype. However, this was not observed; only 13 transcripts were differentially expressed between opioid users and nonusers (*P*_Bonferroni_ < 0.05; Supplemental Figure 10), and the neutrophil degranulation pathway was not enriched, implying that neutrophil degranulation more likely explains the differences between phenotypes than opioid use. In our replication cohort, opioid consumption was linked with increased odds of pain chronicity, in line with previous results. Hence, these combined findings suggest that rhythmic pain intensity and neutrophil degranulation may be key to distinguishing pain profiles and opioid use in CLBP.

The neutrophil degranulation gene signature was identified as a key contributor to pain rhythmicity in our study. Previous work from our group and others has shown an important role for neutrophils in both acute and chronic pain states (34, 58, 64, 65). Upon activation, these cells are recruited to sites of injury and can also infiltrate the central and peripheral nervous systems (34, 65), secreting inflammatory mediators that can act directly or indirectly on peripheral/central neurons, glia, and other immune cells to regulate the onset, maintenance, and resolution of pain (18, 34, 66). A recent meta-analysis of the neutrophil response found these cells to be important contributors to acute and inflammatory pain, though less is known about their effect in chronic pain (64); a recent study using a translational model of fibromyalgia, however, found that recruitment of these cells into the dorsal root ganglia is responsible for peripheral nerve sensitization and the establishment of chronic widespread pain in mice (64). *S100A8* was among the key genes identified in the degranulation response in our dataset. Surprisingly, our previous findings showed that *S100A8* (and *S100A9*) were differentially expressed among patients transitioning from acute to chronic pain. Whether, and how, neutrophils affect pain remain a matter of debate, particularly since the methods most often used for their depletion have been shown to be ineffective for chronic use (67). Further complicating matters is the identification of multiple neutrophil subsets in chronic diseases. Thus, additional preclinical, clinical, and translational studies are needed to better define the contribution of neutrophils and their mediators in both acute and chronic pain.

Results from our main cohort highlight that people with rhythmic pain do not use opioids and are less likely to show a neutrophil activation gene signature compared with the nonrhythmic groups. This may suggest an overactive neutrophil response in those with nonrhythmic pain or a more adaptive immune response in those with rhythmic pain, where neutrophil-derived opioids contribute to pain reduction (35, 66). There is also evidence that opioid use leads to immune dysregulation by compromising both the innate and adaptive immune responses (68). When considered as a proxy of nonrhythmicity in our second cohort (where pain rhythmicity data were not available), opioid use was associated with increased neutrophil activation over time, similar to the nonrhythmic patients in our main cohort. This prolonged, dysregulated, and/or increased neutrophil activation may result in compromised degranulation, inflammation, and endogenous opioid production. Over time, this may contribute to the increased likelihood of nonresolving and chronic pain as the immune system seeks to maintain homeostasis (18). Additionally, our study highlights the importance of pain rhythmicity and neutrophil degranulation over 24 hours, which may be essential when thinking about the role of the immune system in pain regulation, the transition from acute to chronic pain, and perhaps even opioid dependence. Further studies are needed to disentangle these mechanisms, better understand the directionality of effect, and identify specific neutrophil-derived mediators contributing to these outcomes.

There were some limitations to consider in our study. Despite our sample being equally distributed in terms of sex, there was a lack of diversity in racial and ethnic representation, which may limit the generalization of our findings. Given the impact of genetic ancestry on immune function (69, 70), further study of pain and immune cell rhythmicity across diverse populations is necessary. While all participants in our main cohort presented with CLBP with no other competing pain conditions, the underlying cause of CLBP was not investigated. This could be a confounding variable in terms of rhythmicity, as some conditions affect areas of the body that may be influenced by progressive ambulatory activity throughout the day (71). Our cohort was limited by our methodology that integrated EMA data with intraday blood collection to capture circadian changes in pain; this potentially results in underpowered regression analyses. However, MLR was used to avoid multiple testing biases. Our strategy for grouping participants in phenotypes was appropriate for our sample size and effectively divided patients into groups. Alternative approaches (e.g., unbiased methods) should be considered for larger samples. Our EMA e-diary included 3 time points to minimize participant burden, but increased sampling frequency could offer more detailed information about circadian rhythms of pain (9, 43). MLR analyses showed an unconventionally small *P* value for opioid use, which might have been related to the data being skewed with none of the rhythmic group taking opioids. However, the significance of opioid use in our cohort was also detected using Fisher’s exact test. Additionally, our transcriptomics dataset was processed in 2 sequencing batches, ultimately affecting the raw transcript counts; we mitigated this bias by including sequencing batches as a covariate in our analyses. In our replication cohort, we used the international definition that defines chronic pain as persistent for 3 months (72). However, we acknowledge that in clinical practice this may not always reflect the patient experience, with some patients requiring longer treatment to resolve their pain (72, 73). Finally, control healthy subjects without pain were not studied, preventing the comparison of transcriptomes of people who resolved pain to those never experiencing pain.

In summary, we provide an approach using inter- and intraindividual variability of pain rhythmicity and neutrophil degranulation to identify biomarkers of CLBP that can be used to group patients with pain and potentially other chronic conditions. Fluctuations in pain intensity and molecular changes in immune cells over time, likely linked to circadian rhythmicity, are important to consider when treating pain. These strategies can help tailor interventions to specific pain phenotypes. Restoring circadian rhythmicity in those with arrhythmic phenotypes may present an innovative avenue to improve overall outcomes in chronic pain.

## Methods

### Sex as a biological variable

Our study included 62 participants: 35 identified as female and 27 as male. We accounted for sex in all our analyses (transcriptomics and psychosocial) and for both sex and gender in our psychosocial analyses.

### Study design and participants: CLBP cohort

Our cohort included a sample of 74 adults referred to the Kingston Health Sciences Centre (KHSC) Chronic Pain Clinic by their family physicians for CLBP. Recruitment occurred between July 2018 and February 2020. Patients were screened for eligibility during triage (aged 18 years or older, Internet access at home, and experiencing CLBP with persisting symptoms for at least 3 months and half of the days in the past 6 months). Patients with competing pain conditions, untreated sleep disorders, and chronic inflammatory diseases and those working night shifts up to 6 months prior to study enrollment or using melatonin supplements were excluded.

After providing written informed consent, participants completed an initial assessment based on an adapted version of the Canadian CLBP Minimal Registry Dataset (74), designed to capture biopsychosocial characteristics that may affect pain experiences (duration, severity, spread, history of operation, and treatments), medical history (e.g., medication usage and comorbidities), and sociodemographic data. Participants also completed validated questionnaires including the Patient Reported Outcome Measurement Information System (PROMIS-29 v2.0) (75), Pain Catastrophizing Scale (PCS-6) (76, 77), Short-Form Health Survey (SF-12) (78), and PainDETECT Questionnaire (79). Established 11-point numeric rating scales were used to measure intraday changes in pain intensity, depressive mood, and overall fatigue over 7 days. Symptom diaries were administered using Qualtrics, with EMA used to identify temporal variations in pain intensity (9, 43). Participants were prompted to complete their e-diary 3 times per day at 8:00, 14:00, and 20:00; all data were time-stamped, and only those collected within ±1 hour were analyzed.

Our primary objective was to determine whether CLBP patients could be grouped based on intraday self-reported pain intensity, as assessed using data from EMA diary reports over 7 days. Second, we sought to determine if the heterogeneous experience of pain is associated with specific molecular, cellular, and psychosocial phenotypes by analyzing data from the initial assessment and 2 blood samples collected within 12 hours.

### Transcriptome analysis of whole blood

Two tubes of blood were collected twice 12 hours apart (7:00–9:00 and 19:00–21:00) by venipuncture in either arm after completion of the initial assessment. A 4 mL Vacutainer K2 EDTA tube (Becton Dickinson) was used for complete blood count with differential (performed at KHSC). A 3 mL Tempus Blood RNA tube (Applied Biosystems) was used to collect stabilized whole-blood RNA to assess diurnal changes in gene transcription among peripheral immune cells. RNA was extracted using Tempus Spin RNA Isolation kits (Invitrogen) according to the manufacturer’s protocol. RNA integrity (≥7.5) and concentration were assessed prior to library preparation, with globin clearance. RNA sequencing was carried out using the DNBseq platform (BGI Genomics) using 150 bp paired-end reads with approximately 60 million reads per sample. BGI used SOAPnuke v1.5.2 with the parameters “-l 15 -q 0.2 -n 0.05” to clean raw sequencing reads. Data were shipped via physical hard drive and stored by the Queen’s University Centre for Advanced Computing. This sequencing process occurred in 2 batches: the first batch had 64 samples, and the second had 56 samples, 4 of which were secondary aliquoted from the subjects in the first batch.

Using the same samples, qPCR was performed specifically for the following transcripts: qPCR was performed with Bmal1 (Hs00154147_m1), *CLOCK* (Hs00231857_m1), *PER1* (Hs00242988_m1), *PER2* (Hs00256144_m1), and *PER3* (Hs00213466_m1) as gene targets of interest (Thermo Fisher Scientific). Gene-specific master mixes were made by combining 1 μL of the probe specific to the target gene with 10 μL of TaqMan Fast Advanced Master Mix (Applied Biosystems) and 8 μL of diethyl pyrocarbonate–treated water. Using a 96-well MicroAmp Fast Optical Reaction Plate (Applied Biosystems), 10 ng of cDNA was added to each well with 19 μL of master mix. Samples underwent qPCR using a QuantStudio 12 K Flex Real-Time PCR system (Applied Biosystems), where an initial incubation for 2 minutes at 50°C was followed by a brief incubation at 95°C for 20 seconds. The samples then underwent 40 amplification cycles of 1 second at 95°C and 20 seconds at 60°C. All samples were run in either duplicate or triplicate to minimize large differences in Ct between wells. The 2^–ΔΔCt^ results are reported as fold changes in gene expression (80).

### Code availability

No custom code was used to generate or process the data. However, scripts used to analyze the RNA sequencing data are available at https://github.com/ComputationalGenomicsLaboratory/clbpHum (commit ID 87e3368).

### Statistics

#### Theoretical framework for psychosocial analyses.

Differences in sociodemographic factors between groups were analyzed using Fisher’s exact test (categorical) and Kruskal-Wallis test (continuous). Average pain intensity scores across individual time points (8:00, 14:00, and 20:00) or over 7 days and percentage change in immune cells were analyzed using an ordinary 1-way ANOVA with post hoc Tukey’s test among the groups (Figure 1, B and C). All statistical analyses were performed using R v4.3.2, SPSS v0.1.0, or GraphPad Prism v10.0.0 where appropriate.

Multinomial logistic regression analysis (reference category: rhythmic↑) was used to determine the relationship between observed pain phenotype and biopsychosocial characteristics (Figure 2 and Supplemental Figure 5). The risk of belonging to a phenotypic group for a given characteristic was compared with the rhythmic↑ phenotype and expressed as OR. First, we included sex and age in our model as confounding variables. We also assessed pain intensity, pain interference, physical function, depression, sleep disturbance, and catastrophizing as representative measures of key CLBP self-report domains to be included as covariates, as recommended by the NIH Task Force; PROMIS measures offering length-balanced psychometric validity were used where possible. We also considered the effect of medication usage by including pain medication and opioid use in the multinomial logistic regression model. OR and its corresponding 95% CI were reported (Figure 2). MLR was also run to include covariates in our model (Supplemental Figure 5).

#### Transcriptomic analysis of the CLBP cohort.

An analysis workflow for RNA sequencing data is summarized in Supplemental Figure 13. Our pipeline used select tools to perform quality control of sequencing reads, alignment to the reference genome, quantification of reads into transcript abundance counts, and analysis in relation to self-reported pain rhythmicity. Unless otherwise specified, all analyses conducted with R packages were completed in R v3.6.0. We used FastQC v11.9 and MultiQC v1.12 to determine that no further cleaning was needed (81, 82). RNA sequencing reads were then aligned to GENCODE’s primary human reference genome vGRCh38.p13 using Hisat2 v2.2.1 (83, 84) with the parameters “--dta --sensitive --no-discordant --no-mixed” and a mean overall alignment rate of 96.48% (Supplemental Figure 14). Samtools v1.10 was used to sort resulting BAM files, mark duplicates, and index BAM files (85). StringTie v2.1.5 assembled aligned reads into transcripts and quantified their abundance using GENCODE’s vGRCh38.p13 comprehensive gene annotation (86). IsoformAnalyzeR v1.17.4 in R v4.2.1 assigned a known gene to “novel” transcripts based on their genomic coordinates when possible (87). The result of this workflow is a transcript count matrix (*n* = 336,781) for each patient and time point.

Sample outlier detection and trimmed mean of M values normalization of transcript counts were performed using the R packages arrayQualityMetrics v3.42.0 and edgeR v3.28.1 (88,89). arrayQualityMetrics uses 3 metrics to consider a sample an outlier: (a) its sum of distances to other samples, (b) the Kolmogorov-Smirnov statistic, and (c) Hoeffding’s *D* statistic. Samples are removed if marked an outlier before and after normalization or if multiple metrics marked them an outlier after normalization. No samples met these criteria. We also used variance stabilizing transformation (VST) and principal component analysis with the R package DESeq2 v1.26.0 to visualize sample clustering (Supplemental Figure 15) (90). We applied nonspecific filtering to normalized transcript counts based on their median absolute deviation and kept the top 30% variable transcripts (*n* = 101,035; Supplemental Figure 15) for downstream analyses. For network analysis, we used ComBat-seq from sva v3.36.0 in R to adjust raw and filtered transcript counts for batch effects (Supplemental Figure 15) (91, 92). Patients’ sex was obtained from electronic medical records and confirmed using expression of sex-specific genes *XIST* and *SPRY*. Finally, we applied VST to the adjusted counts.

Differential expression of transcripts was analyzed to identify univariate differences between the rhythmic↑ phenotype and other phenotypes. Using R package edgeR’s GLM adjusted for sequencing batch (1/2), sex (male/female), and time point (day/night), we first asked which transcripts differ between the rhythmic↑ and any other phenotypes. Then, we compared the expression of transcripts between the rhythmic↑ and constant-low, constant-high, and mixed phenotypes, individually. Significant transcripts had Bonferroni-adjusted *P* values < 0.05. Candidate differential expression analysis was also performed using this methodology to investigate the expression of transcripts corresponding to the Gene Ontology pathways GO: 0002544, GO: 0050729, GO: 0070942, GO: 0042119, GO: 0043312,GO: 0043313, GO: 0002283, GO: 0030223, GO: 0030593, GO: 0001780, and GO: 0002446 (Supplemental Figure 9). The R package gprofiler2 v0.2.1 was used for pathway enrichment analysis of DETs (93, 94). Pathway analysis results were subset by adjusted *P* value < 0.05, term size ≥ 10, and term size ≤ 500.

We used WGCNA v1.70-3 in R to identify multivariate differences between the rhythmic↑ phenotype and other phenotypes at the transcript level (95). To minimize bias risk from paired samples, we constructed a signed network for both the day and night samples separately. First, WGCNA calculated the Pearson’s correlation between transcripts using their batch-adjusted and VST counts. Next, correlations were transformed into an adjacency matrix, which encoded the connection strength between transcripts. This adjacency matrix was calculated by raising the correlation values to a power β, hence making weak connections weaker and strong connections stronger. The optimal β to achieve a network with a scale-free topology was determined using the pickSoftThreshold function. Using this approach, we used β = 12 for the day network and β = 12 for the night network (Supplemental Figure 16). Once networks were constructed, we could identify groups of highly connected transcripts called modules. First, we calculated topological overlap matrices (TOMs), which accounted for the adjacency between 2 transcripts and their number of shared connections. These TOMs were converted to dissimilarity matrices (1-TOM) and input to hierarchical clustering. Branches of hierarchical clustering dendrograms represented modules and were identified using the Dynamic Tree Cut method. Then, WGCNA performed principal component analysis to calculate a representative eigengene for each module. Finally, modules were iteratively merged until no module’s eigengene had a 30% or greater Pearson’s correlation with another module (Supplemental Figure 16).

Finally, we calculated the association between transcript clusters and the rhythmic↑ phenotype. We used these eigengene values to find the association between modules and the rhythmic↑ phenotype, either as a binary or multilevel factor. When pain phenotype was a binary, we used a GLM to assess association. For the multilevel factor approach, we used a multinominal GLM from the nnet R package (96). Significant associations had a nominal *P* value < 0.05. Pathway enrichment analysis was performed on modules with significant associations, and results were subset as previously described.

#### Transcriptomic replication cohort.

A previously published transcriptomics study of LBP (Gene Expression Omnibus [GEO]: GSE177034) in which information about drug consumption was tracked in 97 participants was used to replicate our findings. Study participants with significant acute back pain (≥4 on the NRS pain scale) were recruited at the first pain assessment visit and reevaluated 3 months later. Blood samples were taken at first and follow-up visits for transcriptomic analysis as previously described (18); blood samples were not taken at any specific time of the work day. The OR for pain chronification at the follow-up visit (yes/no) upon opioid consumption (yes/no) was estimated from a logistic regression considering the participant’s sex (male/female), age (quantitative), and smoking status (yes/no). OR was obtained from the regression effect (*β*) via OR = e*^β^* and 95% CI from the effect SEM via e^(*β*±1.96.sem)^. We first performed an analysis to detect differentially expressed genes (DEGs) between opioid users and nonusers. Gene expression was regressed using an opioid use × time interaction term, asking whether the expression time trajectories of a gene differed between those who consumed opioids during the study and those who did not, and corrected for age (quantitative), sex (male/female), sample RNA integrity number (RIN; quantitative), and smoking status (yes/no). Analyses were further corrected for pain outcome at the follow-up visit as a binary variable, as we previously showed that active neutrophil processes were involved in pain resolution (affecting the likelihood of chronification; yes/no). The interaction term analyses required each interaction’s variable to appear by itself and so was further corrected for opioid consumption (yes/no) and for time point (baseline/follow-up). Overall, the design equation in R package DESeq2 (90) was design ~age + sex + RIN + smoker + resolved + time + opioids + time × opioids. The mathematical sign of the interaction term following the regression indicated how the gene’s time trajectory between the 2 visits in those consuming opioids contrasted with those that did not: a positive sign indicated that the gene expression in opioid consumers lingered or was higher than those not consuming opioids (counterclockwise rotation). We performed a pathway analysis based on DEG results using the R package fgsea, where all genes were considered but only those that were coregulated within a pathway contributed to its significance. Genes were then ranked from most positively to most negatively interacting for input to fgsea for pathway analyses in Gene Ontology’s biological processes (GO:BP) (97). Specifically, we focused on hematopoietic cell activation and degranulation processes. fgsea also provided pathway enrichment score plots for specific pathways. *P* values were reported as uncorrected or corrected for FDR where appropriate.

### Study approval

Our study was approved by the Queen’s University Health Sciences and Affiliated Teaching Hospitals Research Ethics Board (file 6022651), and all patients provided written informed consent to participate in the study.

### Data availability

RNA sequencing data are available in GEO for the CLBP main cohort (GSE295758) and for the LBP cohort (GSE177034). Values for all data points in graphs are reported in the Supporting Data Values file. Additional information, raw data, and materials are available upon reasonable request.

## Author contributions

The order of the co–first authors was determined on the basis of the significance, time, and effort that each author invested in the project. NG, QD, and LD conceived the project. DT, AMZ, HGMG, and MK developed methodology. EJB, LNS, JDC, MC, ZL, and MGP contributed to methodology. DT, AMZ, HGMG, MK, and MP performed data analysis. ZYF and SAS contributed to the data analysis. MK, EJB, EB, DC, and RW contributed to data acquisition, clinical sample collection, and analysis. NG, DT, AMZ, and HGMG wrote the manuscript. All co-authors contributed to the draft.

## Supplementary Material

Supplemental data

ICMJE disclosure forms

Supporting data values

## Figures and Tables

**Table 2:**
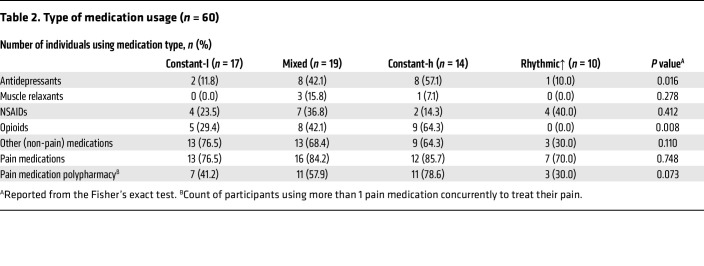
Type of medication usage (*n* = 60)

**Table 1:**
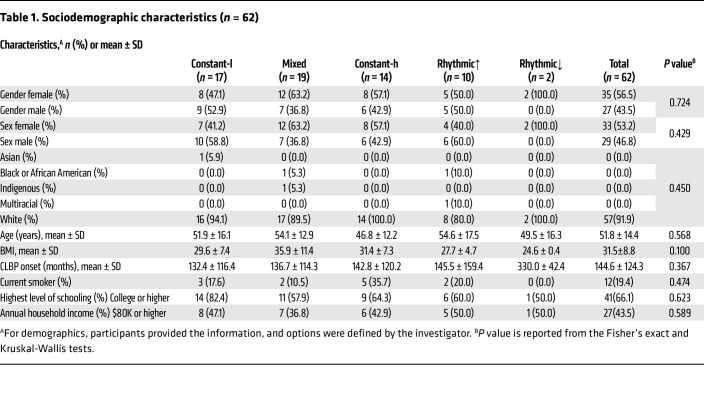
Sociodemographic characteristics (*n* = 62)

## Version 1 10/01/2025

Electronic publication
