# Supplementary material for: Circadian rhythmicity and biopsychosocial characteristics influence opioid use in chronic low back pain
Source: J Clin Invest. 2025 Oct 1;135(19):e188620. doi: 10.1172/JCI188620 (PMC12483613; doi:10.1172/JCI188620)
Supplement: Supplemental data [file jci-135-188620-s311.pdf]

Supplemental material

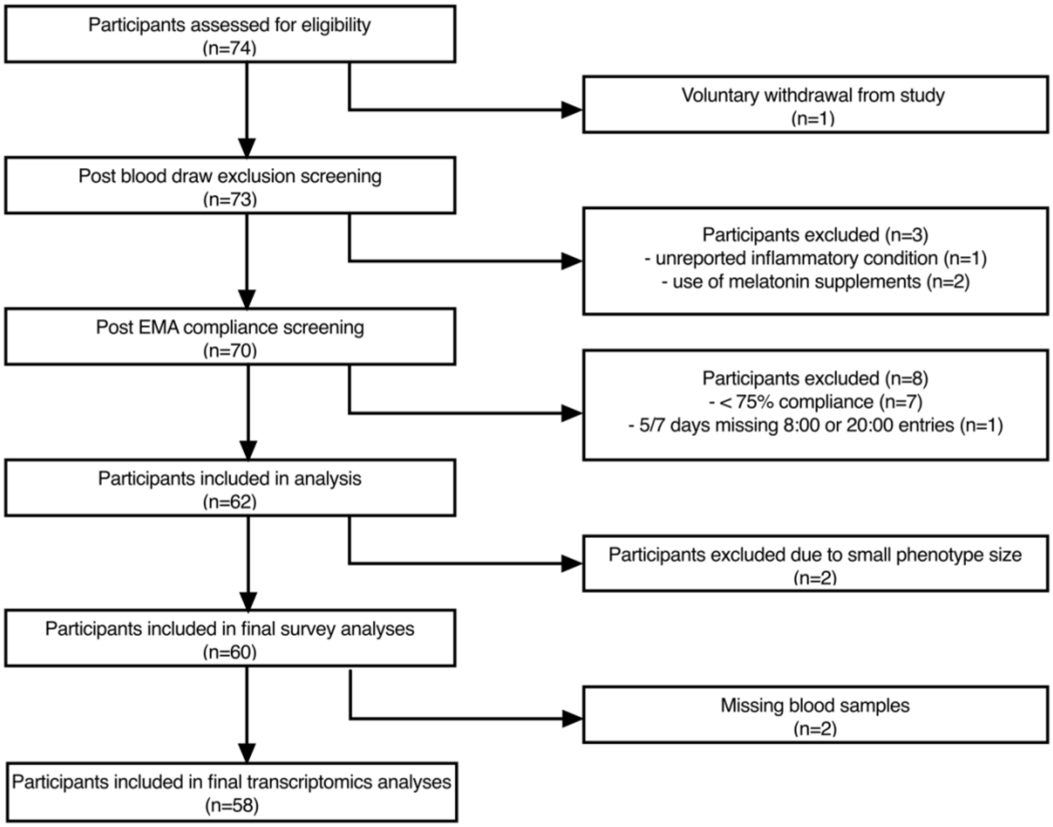

Supplemental Figure 1: Study flowchart of participants based on inclusion and exclusion criteria.

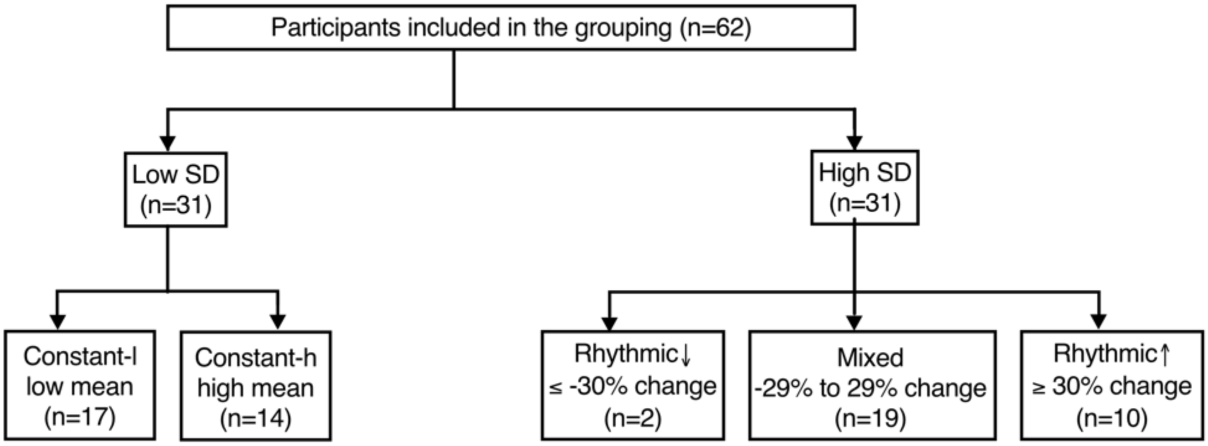

Supplemental Figure 2: Study flowchart of pain phenotypes. Electronic EMA sampling was used to collect data regarding current pain intensity from all participants at 8:00, 14:00, and 20:00 over a 7-day period. Group division based on pain intensity was carried out using a 50% threshold in standard deviation (SD) and a combination of methods including mean and variation in intensity submitted throughout the sampling period. Individuals with high weekly variation were further grouped based on the percent change between scores submitted at 8:00 and 20:00. A 30% change in score is representative of a minimal clinically relevant change in pain intensity. The rhythmic↓ pain group (n=2) was excluded from further analyses due to its small sample size.

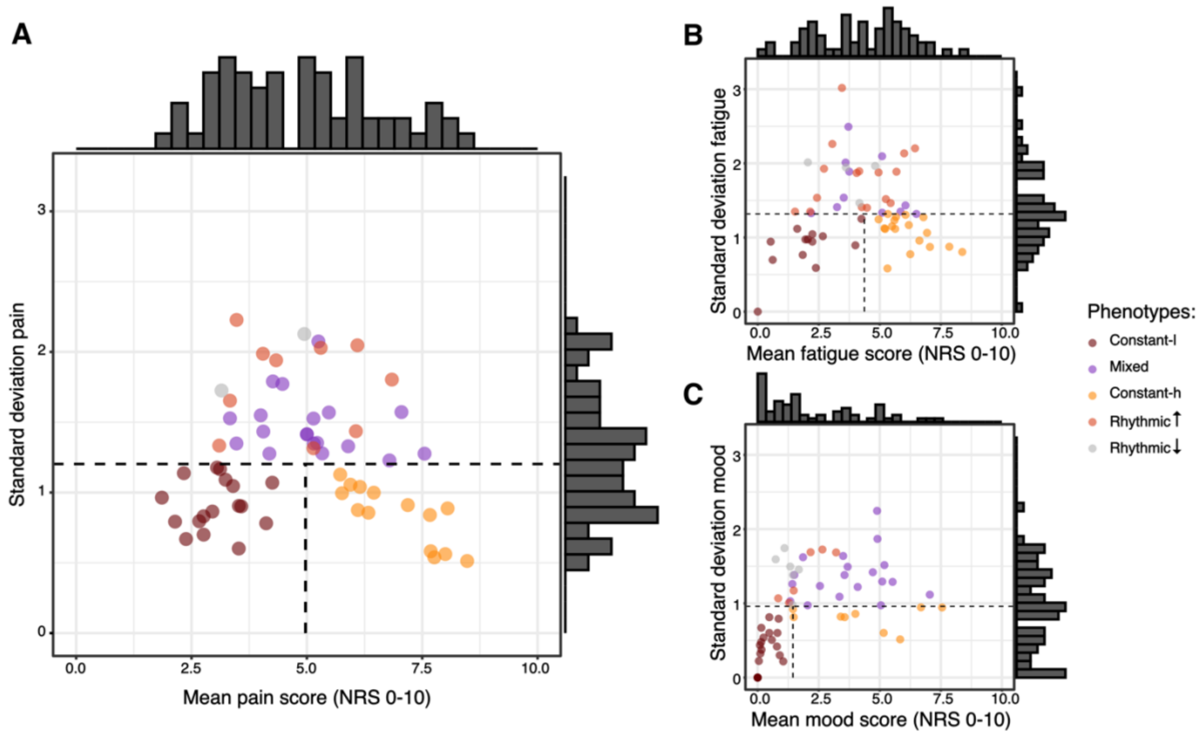

Supplemental Figure 3: Distribution of mean and SD in symptom intensity throughout the sampling period. Mean and SD in self-reported (**A**) pain intensity, (**B**) fatigue intensity, and (**C**) depressive mood throughout the week-long EMA sampling period were calculated for each individual; each point is representative of a single participant. The 50th percentile threshold was used to dichotomize the sample into low and high SD groups. Based on mean pain intensity and percent change in pain score between morning and evening timepoints, participants were then further classified as having constant-l, mixed, constant-h, and rhythmic↑ pain phenotypes and are shown in brown, purple, orange, and red, respectively (**A**). Two participants classified as having rhythmic↓ pain are indicated in grey and were excluded from further analyses due to the low sample size. The same parameters were used to divide participants based on their fatigue (**B**) and mood (**C**) scores, into constant-l, mixed, constant-h, rhythmic↑, and rhythmic↓ phenotypes.

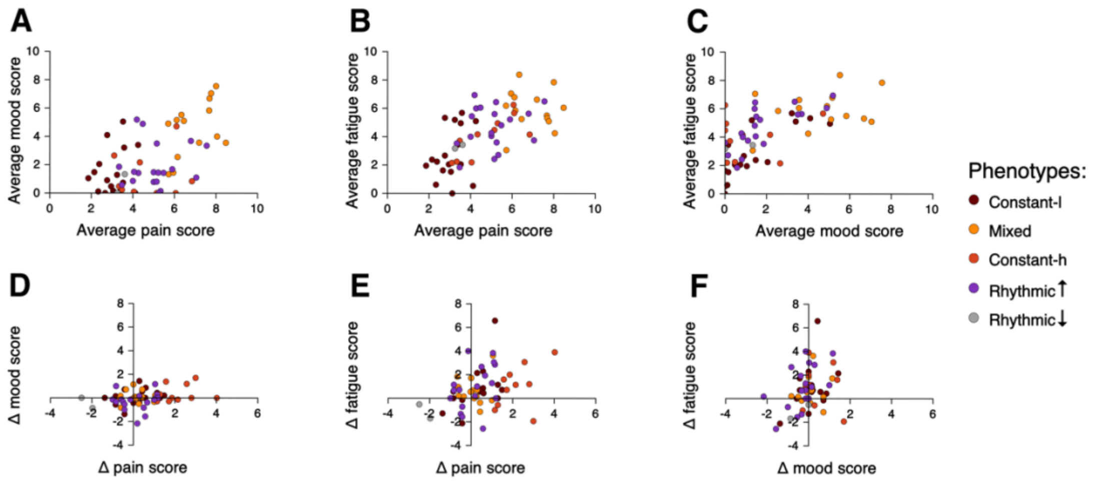

Supplemental Figure 4: Correlation of pain, mood, and fatigue scores. Correlation of self-reported pain, mood, and fatigue intensity via EMA were assessed using the (**A, B,** and **C**) average of these scores across all timepoints and the (**D, E,** and **F**) average change in these scores between 8:00 and 20:00. There were statistically significant, moderate to strong correlations for mean daily scores for pain with mood (**A**: r_s=0.479, P<0.0001) and fatigue (**B**: r_s=0.659, p<0.0001), as well as mood and fatigue (**C**: r_s=0.672, P<0.0001); such strong correlations were not apparent when comparing mean daily change in scores between these measures (**D**: r_s=0.300, P=0.0178; **E**: r_s=360, P=0.0041; **F:** r_s=0.320. P=0.0113). Assessed by Spearman's rank correlation coefficient.

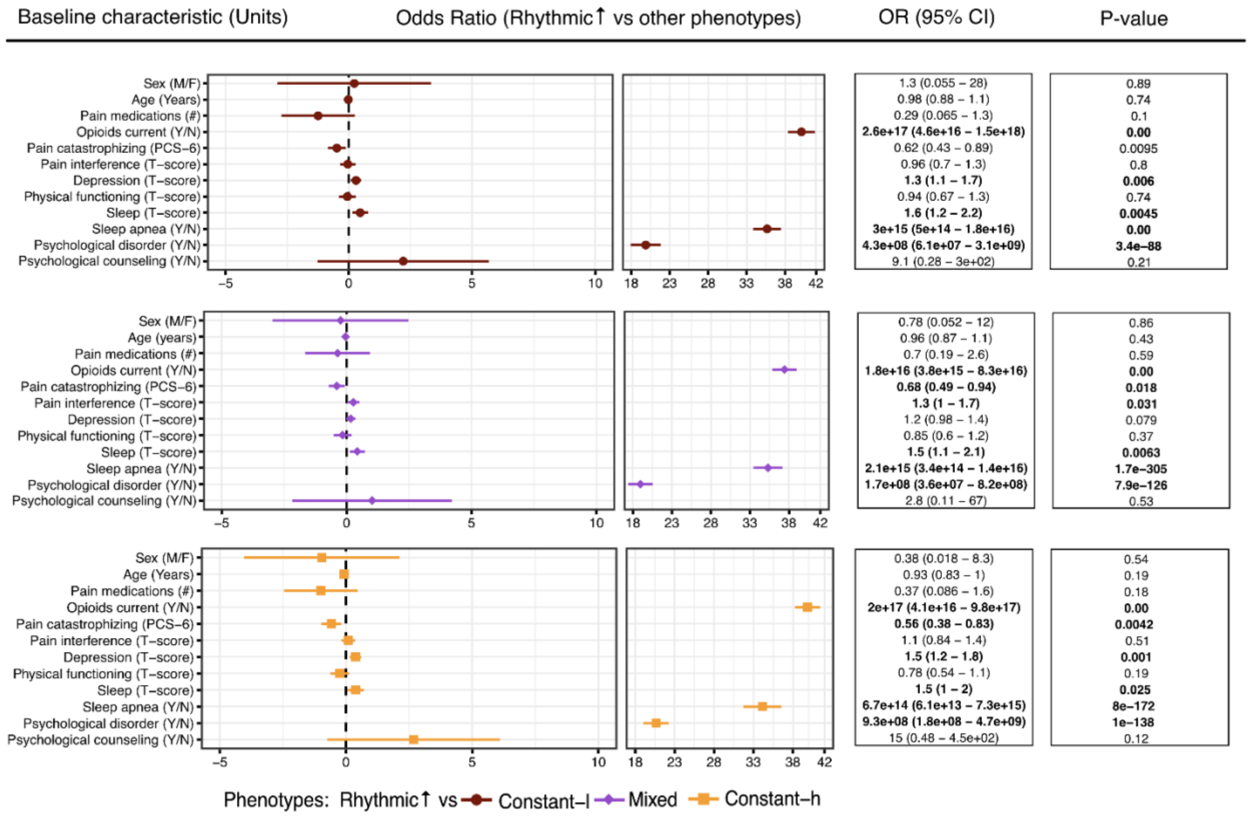

Supplemental Figure 5: Biopsychosocial measures from the CLBP cohort differ across grouped pain phenotypes (n=60). Multinomial logistic regression of psychosocial variables. The number of pain and opioid medications, presence of sleep apnea, psychological disorder, and psychological counseling are included in the medical history questions. Sleep disturbance, pain interference, physical functioning, and depression are measured using PROMIS-29 v2.0 scales, for which results are represented in t-score, where 50 is indicative of the score in an average general American population. A 10-point difference in t-score represents a change of one SD difference in the severity of the assessed behavioral factors. Pain catastrophizing was measured with PCS-6, range 0–24; a higher score indicates a greater degree of pain catastrophizing. Plotted on the x-axis are the regression coefficients β (log odds). Odds ratios (OR) are reported as numbers with 95% confidence intervals in parentheses. P-values are reported as numbers. Significant results are bolded (P<0.05).

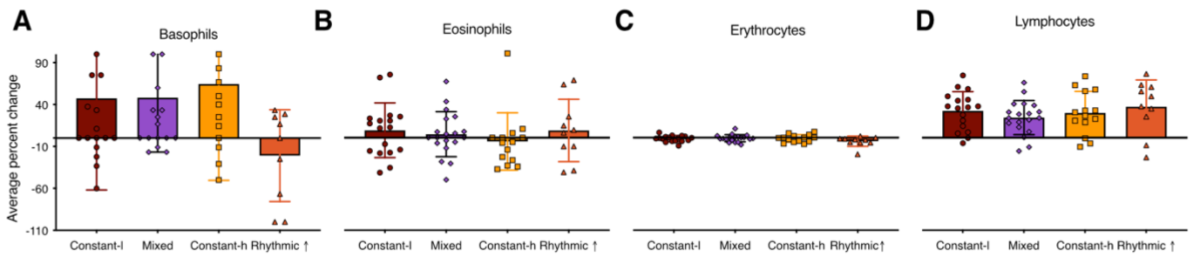

Supplemental Figure 6: Complete blood cell count with differential was performed on peripheral blood collected at 8:00 and 20:00 and the percent change in cell quantity was calculated. No significant differences were observed in percent change of basophils (**A**: P=0.1709)**,** eosinophils (**B**: P=0.6622)**,** erythrocytes (**C**: P=0.2129), and lymphocytes (**D**: P=0.5643). Data were analyzed using a one-way ANOVA with post hoc Tukey test. Data are represented as mean ± SD.

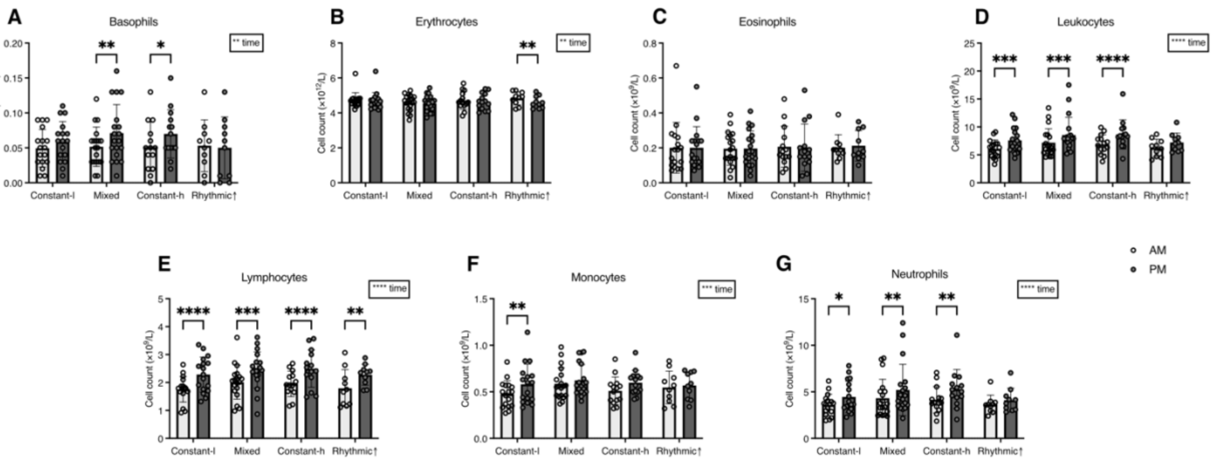

Supplemental Figure 7: Raw values of complete blood cell count with differential performed on peripheral blood collected at 8:00 and 20:00. Basophil count (**A**) was found to be significantly higher at 20:00 than 8:00 in both mixed and constant-h phenotypes (interaction, P=0.1388; pain phenotype, P=0.8056; time, P=0.0025; mixed 8:00 v 20:00, P=0.0088; constant-h 8:00 v 20:00, P=0.0443). The count of erythrocytes (**B**) was significantly different between timepoints within the rhythmic↑ phenotype (interaction, P=0.1828; pain phenotype, P=0.7566; time, P=0.0013; rhythmic↑ 8:00 vs 20:00, P=0.0078). No significant differences in eosinophil count (**C**) were observed between phenotypes or timepoints (interaction, P=0.9191; pain phenotype, P=0.9944; time, P=0.8115). Overall leukocyte count (**D**) was significantly increased at 20:00 in the constant-l, mixed, and constant-h phenotypes (interaction, P=0.5427; pain phenotype, P=0.3601; time, P<0.0001; constant-l 8:00 v 20:00, P=0.0001; mixed 8:00 v 20:00, P=0.0002; constant-h, P<0.0001). Lymphocyte count (**E**) was significantly greater at 20:00 across all phenotypes (interaction, p=0.8915; pain phenotype, p=0.5486; time, P<0.0001; constant-l 8:00 v 20:00, P<0.0001; mixed, P=0.0002; constant-h 8:00 v 20:00, P<0.0001; rhythmic↑ 8:00 v 20:00, P=0.0040), whereas monocyte count (**F**) was only significantly increased at 20:00 in the constant-l phenotype (interaction, P=0.2346; pain phenotype, P=0.5117; time, P=0.0005; constant-l 8:00 v 20:00, P=0.0043). The count of neutrophils (**G**) was observed to increase significantly between the 8:00 and 20:00 timepoints in all phenotypes but rhythmic↑ (interaction, P=0.5530; pain phenotype, P=0.4146; time, P<0.0001; constant-l 8:00 v 20:00, P=0.0106; mixed 8:00 v 20:00, P=0.0046; constant-h 8:00 v 20:00, P=0.0015). Data were analyzed using a two-way repeated measures ANOVA with post hoc Bonferroni's multiple comparisons test. Data are represented as mean ± SD. * P≤0.05, ** P≤0.01, *** P≤0.001, **** P<0.0001.

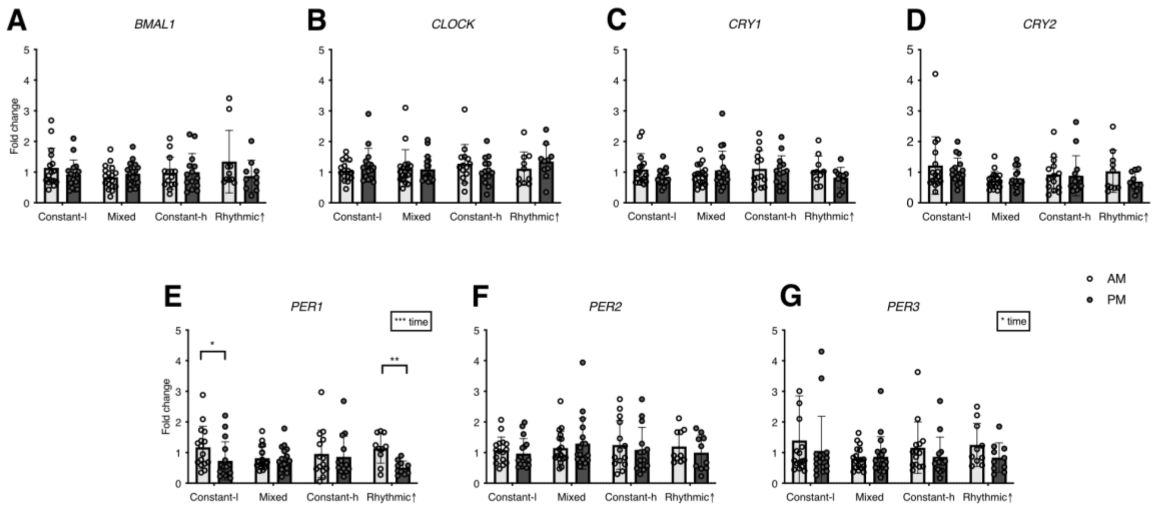

Supplemental Figure 8: Peripheral clock gene expression between 8:00 and 20:00 across pain phenotypes. Gene expression at the mRNA level was assessed by qPCR on peripheral blood samples collected from each participant at 8:00 and 20:00. No significant differences in variation of gene expression were observed between pain phenotypes for (**A**) *BMAL1* (interaction, P=0.1867; pain phenotype, P=0.6128; time, P=0.1738), (**B**) *CLOCK* (interaction, P=0.1523; pain phenotype, P=0.9119; time, P=0.6124), (**C**) *CRY1* (interaction, P=0.1647; pain phenotype, P=0.8574; time, P=0.1486), or (**D**) *CRY2* (interaction, p=0.4393; pain phenotype, P=0.1443; time, P=0.1855). (**E**) *PER1* had a significantly higher level of expression at 8:00 relative to at 20:00 in both the constant-l and rhythmic↑ pain phenotypes, while no other significant differences were observed (interaction, P=0.0341; pain phenotype, P=0.7925; time, P=0.0003; constant-l 8:00 v 20:00, P=0.0143; rhythmic↑ 8:00 v 20:00, P=0.0048). (**F**) *PER2* (interaction, P=0.4975; pain phenotype, P=0.7479; time, P=0.3855), (**G**) *PER3* (interaction, P=0.3715; pain phenotype, P=0.5441; time, P=0.0250). Data were analyzed using a two-way RM ANOVA with post hoc Bonferroni's multiple comparisons test. Data are represented as mean ± SD. * P≤0.05, ** P≤0.01, *** P≤0.001.

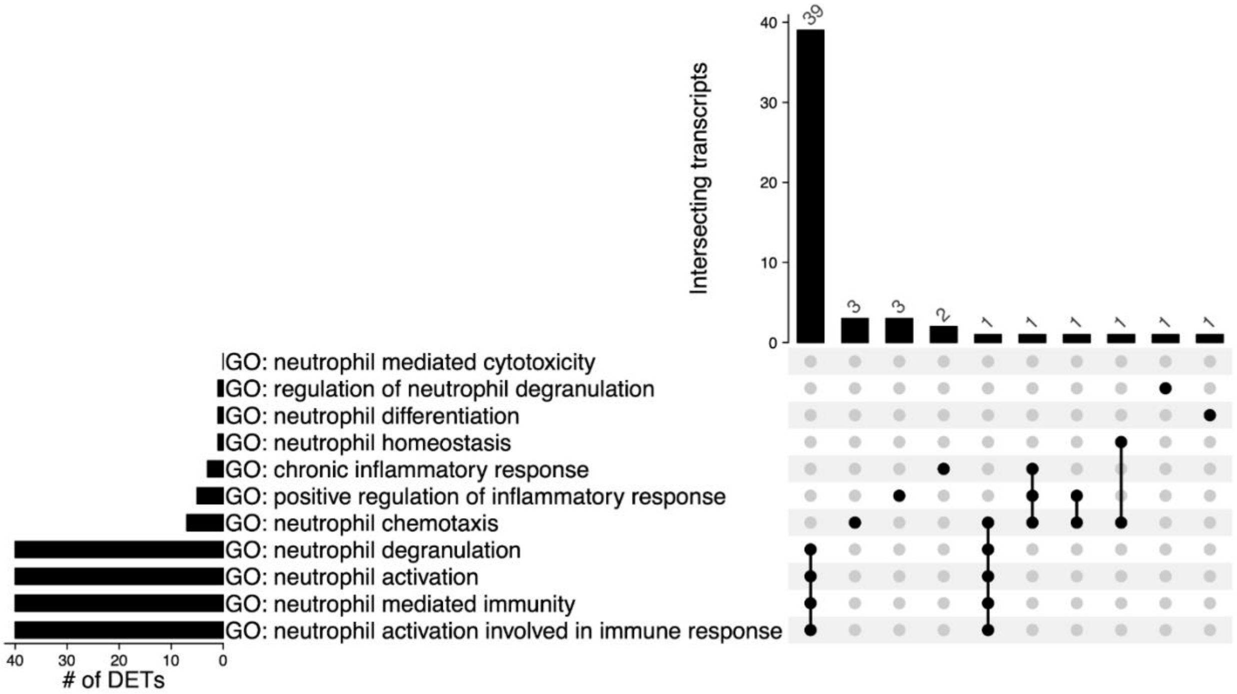

Supplemental Figure 9. Differentially expressed transcripts corresponding to neutrophil-related inflammatory pathways. UpSet plot indicating the number of differentially expressed transcripts (DETs) for each candidate analysis ($P_{Bonferroni}$<0.05) and their intersections across pathways.

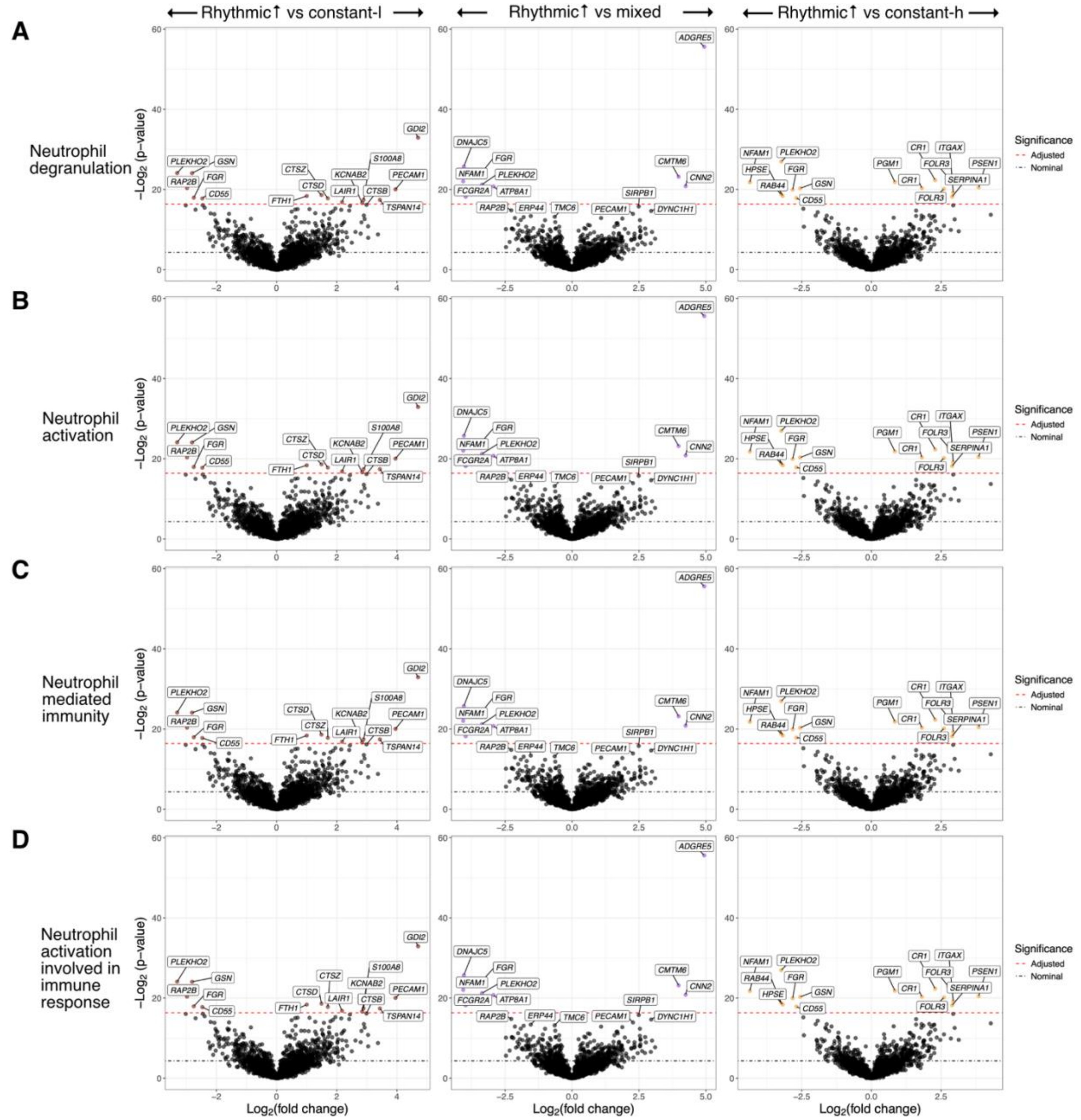

Supplemental Figure 10. Chronic inflammatory mediators differentially expressed across pain rhythmicity phenotypes. Differentially expressed transcripts (DETs, coloured points) between the rhythmic↑ phenotype and other phenotypes ($P_{Bonferroni}$<0.05). The top 15 transcripts are labelled by their respective genes. (**A**) Transcripts found in the neutrophil degranulation pathway (GO:0043312) were tested (n=4153). DETs: constant-l n=14, mixed n=9, constant-h n=15. (**B**) Transcripts found in the neutrophil activation pathway (GO:0042119) were tested (n=4243). DETs: constant-l n=14, mixed n=9, constant-h n=15. (**C**) Transcripts found in the neutrophil mediated immunity pathway (GO:0002446) were tested (n=4254). DETs: constant-l n=14, mixed n=9, constant-h n=15. (**D**) Transcripts found in the neutrophil activation involved in

immune response pathway (GO:0002283) were tested (n=4182). DETs: constant-l n=14, mixed n=9, constant-h n=15.

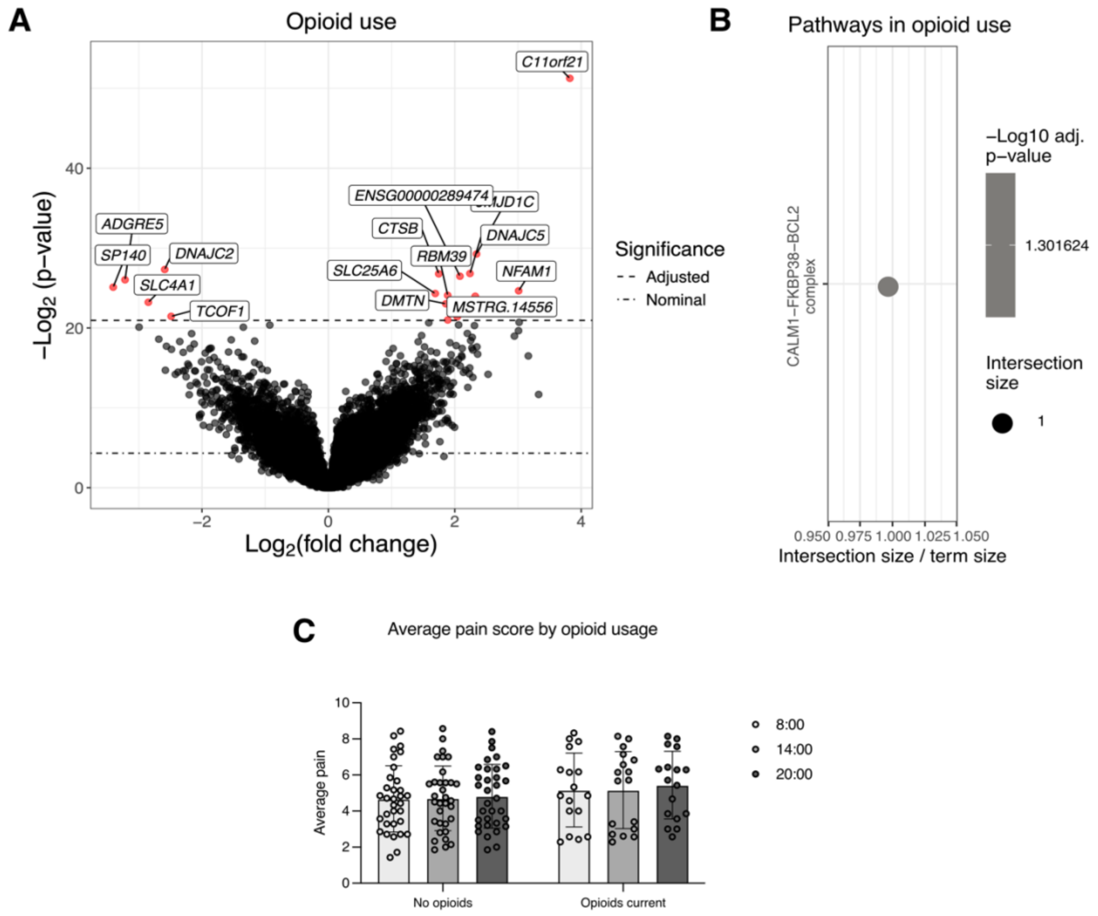

Supplemental Figure 11: Transcriptomic analysis of opioid use among non-rhythmic↑ participants. Differential gene expression between current opioid non-users and users with a non-rhythmic↑ phenotype (**A**). Volcano plots of differentially expressed transcripts (DETs, colored points, n=17) between the current opioid non-users and users ($P_{Bonferroni}$<0.05). The top 15 transcripts are labelled by their respective genes. Pathway enrichment analysis of genes corresponding to DETs (**B**). The top 10 significant pathways ($P_{g:SCS}$<0.05, pathway size≥10 and ≤500) are shown on the y-axis. Average pain score at 8:00, 14:00, and 20:00 among opioid users and non-users with a non-rhythmic↑ phenotype (**C**). No significant differences were identified between timepoints or opioid use groups (interaction, P=0.7681; opioid usage, P=0.3419; time, P=0.1379). Significance was assessed with a two-way repeated measures ANOVA. Data are represented as mean ± SD.

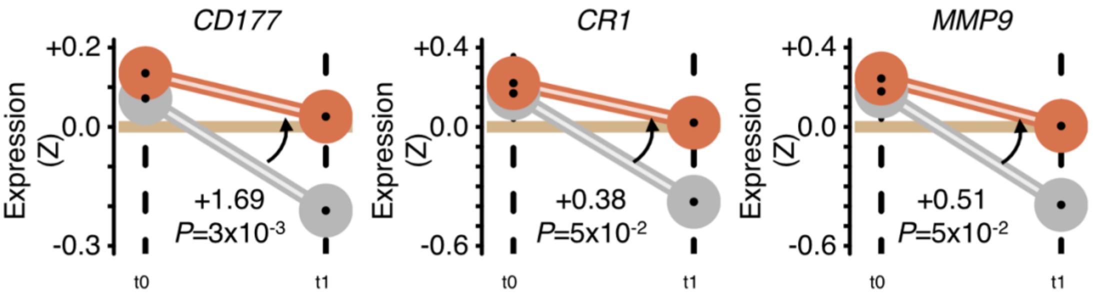

Supplemental Figure 12: Gene expression time trajectories, in those taking opioids (orange) and in those not (grey), in selected genes: (**A**) the CD177 molecule (CD177, also known as the Human Neutrophil Alloantigen, (**B**) the Complement C3b/C4b Receptor 1 (CR1), and (**C**) the Matrix Metallopeptidase 9 (MMP9)**.** Expressions of genes were normalized via Z-scoring (Z) such that y-axes indicate the number of standard deviations. Opioid use × time effect sizes and corresponding but unadjusted P-values are indicated. All effect sizes were positive (black curved arrows), indicating lingering gene expression with time in opioid users compared to non-users.

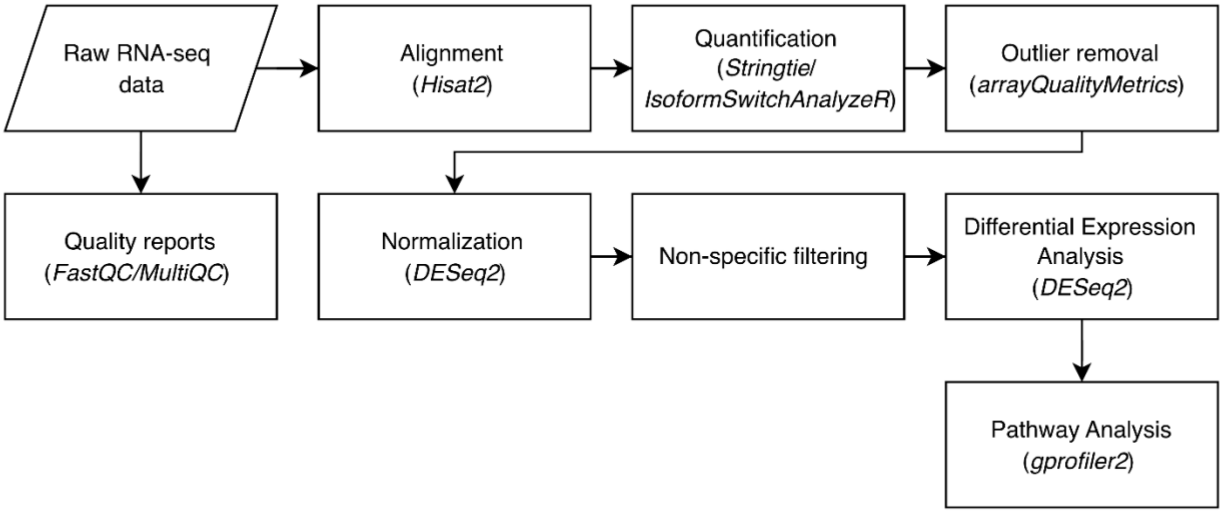

Supplemental Figure 13: Flowchart of the RNA-sequencing data analysis. The Beijing Genomics Institute cleaned the raw RNA sequencing reads with SOAPNuke v1.5.2. We used FastQC and MultiQC to evaluate the quality of sequencing reads and determine that no further cleaning was needed. Hisat2 aligned reads to GENCODE's primary human reference genome vGRCh38.p13. Transcript counts were quantified with StringTie. We used IsoformAnalyzeR to annotate novel transcripts with their respective genes based on their genomic coordinates. We then performed outlier detection and TMM normalization of transcript counts using R packages arrayQualityMetrics and edgeR. arrayQualityMetrics uses three metrics to consider a sample an outlier: 1) its sum of the distance to other samples, 2) the Kolmogorov-Smirnov statistic, and 3) the Hoeffding's D-statistic. Samples are removed if marked an outlier before and after normalization, or if multiple metrics marked them an outlier after normalization. No samples met these criteria. Next, we calculated the variability of each transcript's expression across samples using the median absolute deviation method. The top 30% variably expressed transcripts (n=101,035) were kept. For network analysis, we used ComBat-seq from the sva R package to adjust the raw and filtered transcript counts for batch effects. These adjusted counts were variance stabilizing transformed with DESeq2 for network analysis. For differential expression analysis, we asked what transcripts were differentially expressed between the rhythmic↑ phenotype and others with edgeR's recommended additive generalized linear model (GLM) approach. For network analysis, we used WGCNA to construct a signed transcript co-expression network for the day and night samples separately. We used both a GLM and multinomial GLM to identify transcript clusters associated with the rhythmic↑ phenotype, either as a binary or multi-level factor. Finally, we used gprofiler2 to perform pathway enrichment analysis on differentially expressed transcripts and transcript clusters.

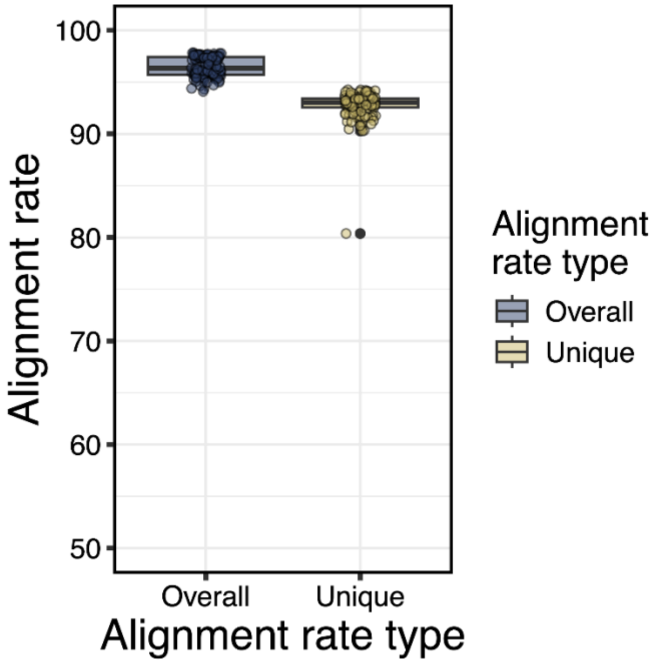

Supplemental Figure 14: Boxplot representing alignment rates of RNA sequencing reads to the human reference genome. The overall alignment rate is the total number of aligned reads divided by the input number of reads times 100. The unique alignment rate is the number of reads that aligned to only one region of the reference genome divided by the input number of reads times 100. Each coloured data point represents the overall (blue) and unique (yellow) alignment rate for a sample.

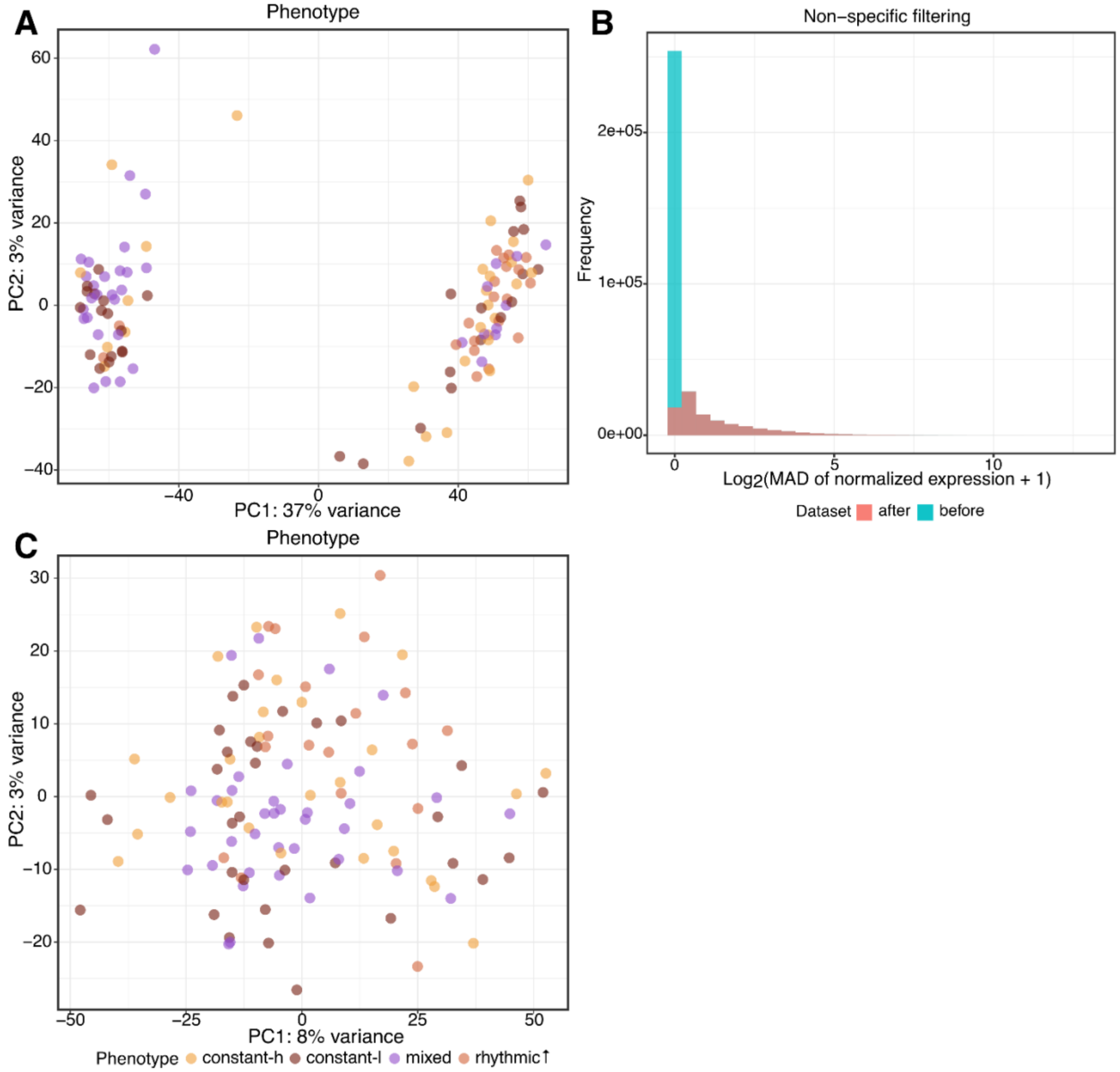

Supplemental Figure 15: Data preparation of RNA sequencing data. (**A**) Principal component analysis of variance stabilizing transformed (VST) transcript counts before data cleaning. Data points represent samples and are coloured by their pain phenotype. (**B**) Distribution of the median absolute deviation of transcripts' counts before and after non-specific filtering. (**C**) Principal component analysis of VST transcript counts after filtering and adjustment for batch effects.

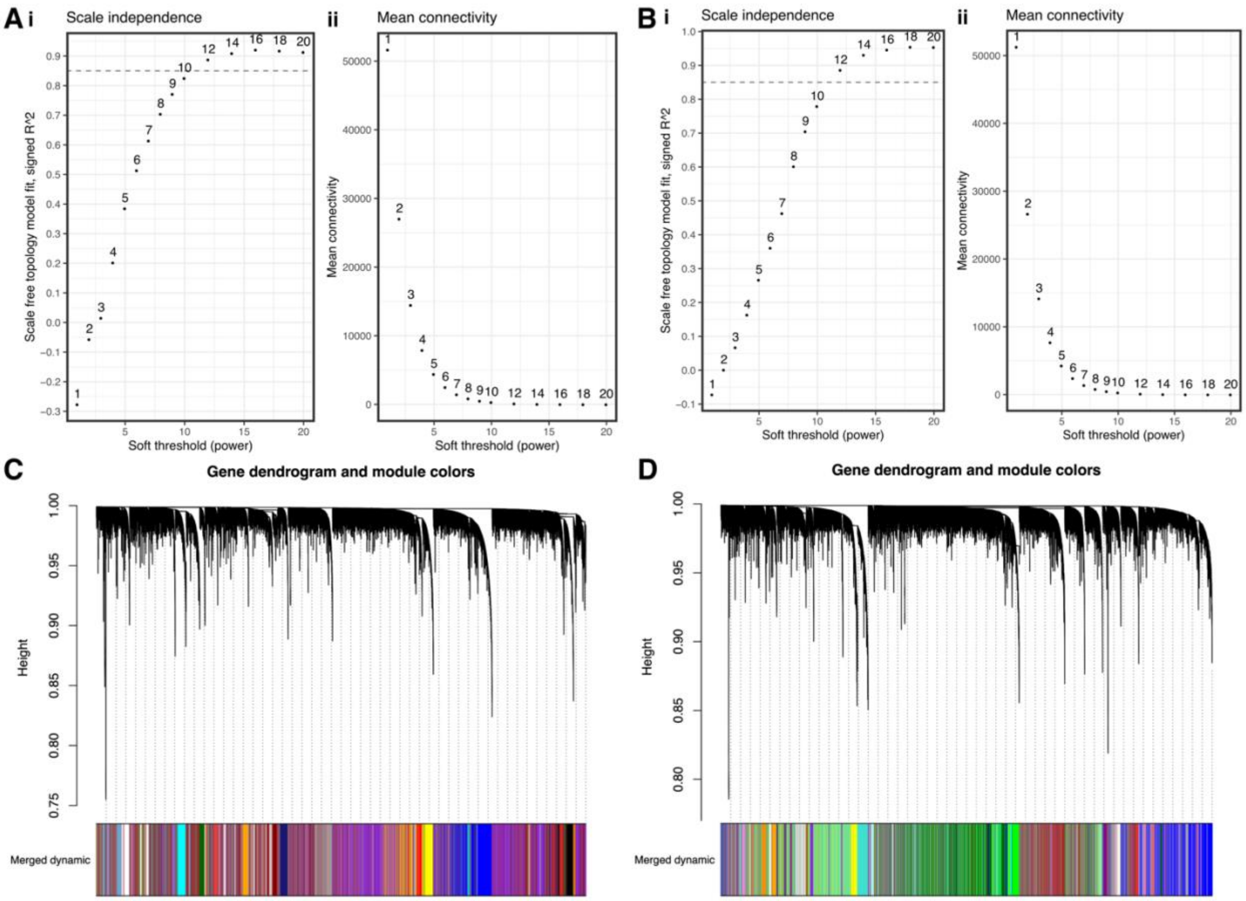

Supplemental Figure 16: Construction of weighted gene co-expression networks for the day and night. (**A** and **B**) Scale independence and mean connectivity plots used to determine the optimal soft thresholds for network construction. (**A**) The day network. (**B**) The night network. (**C** and **D**) Transcript dendrograms illustrating the final clustering of transcripts into modules. (**C**) The day network. (**D**) The night network.

Supplemental Table 1. Medical history (n=60)

Number of participants possessing medical history characteristics, n (%)						
		Constant-l (n=17)	Mixed (n=19)	Constant-h (n=14)	Rhythmic- (n=10)	P-value*
Report LBP every day or nearly every day		14 (82.4)	19 (100.0)	14 (100.0)	8 (80.0)	0.041
Report LBP that has radiated down legs in the past 2 weeks		7 (41.2)	11 (57.9)	13 (92.9)	8 (80.0)	0.124
Bothered by stomach pain in the past 4 weeks	Not bothered	13 (76.5)	10 (52.6)	8 (57.1)	6 (60.0)	0.544
	Bothered a little	4 (23.5)	7 (36.8)	3 (21.4)	4 (40.0)	
	Bothered a lot	0 (0.0)	2 (10.5)	2 (14.3)	0 (0.0)	
Bothered by joint and limb pain in the past 4 weeks	Not bothered	4 (23.5)	0 (0.0)	1 (7.1)	0 (0.0)	0.099
	Bothered a little	8 (47.1)	8 (42.1)	3 (21.4)	4 (40.0)	
	Bothered a lot	5 (29.4)	11 (57.9)	10 (71.4)	5 (50.0)	
Bothered by upper-back, neck, or shoulder pain in the past 4 weeks	Not bothered	7 (41.2)	7 (36.8)	1 (7.1)	3 (30)	0.006
	Bothered a little	8 (47.1)	4 (21.1)	4 (28.6)	6 (60)	
	Bothered a lot	1 (5.9)	8 (42.1)	9 (64.3)	1 (10)	
Bothered by headaches in the past 4 weeks	Not bothered	11 (64.7)	12 (63.2)	5 (35.7)	5 (50.0)	0.256
	Bothered a little	5 (29.4)	6 (31.6)	6 (42.9)	5 (50.0)	
	Bothered a lot	1 (5.9)	0 (0.0)	3 (21.4)	0 (0.0)	
Bothered by widespread pain in the past 4 weeks	Not bothered	11 (64.7)	5 (26.3)	2 (14.3)	6 (60.0)	0.014
	Bothered a little	4 (23.5)	10 (52.6)	4 (28.6)	3 (30.0)	
	Bothered a lot	2 (11.8)	4 (21.1)	8 (57.1)	1 (10.0)	
Has had LB operation before		0 (0.0)	4 (21.1)	3 (21.4)	3 (30.0)	0.455
Has received injection treatment		6 (35.3)	5 (26.3)	5 (35.7)	1 (10.0)	0.216
Has received exercise therapy		17 (100.0)	12 (63.2)	12 (85.7)	9 (90.0)	0.054
Has received psychological counseling		4 (23.5)	4 (21.1)	7 (50.0)	2 (20.0)	0.287
Likelihood of NeP component presence**	Unlikely	9 (52.9)	7 (36.8)	3 (21.4)	4 (40.0)	0.190
	Likely	2 (11.8)	4 (21.1)	8 (57.1)	2 (20.0)	
	Ambiguous/ unclear	6 (35.3)	8 (42.1)	3 (21.4)	4 (40.0)	
Used cannabis		2 (11.8)	3 (15.8)	2 (14.3)	2 (20.0)	1.000

LBP = low back pain; LB = low back; NeP = neuropathic pain
*P-value is reported from the Fisher-Freeman-Halton Exact test
** assessed by PainDETECT questionnaire

Supplemental Table 2. Comorbidities

Number of individuals with comorbidity, n (%)					
	Constant-l (n=17)	Mixed (n=19)	Constant-h (n=14)	Rhythmic- (n=10)	P-value*
Hypertension	7 (41.2)	7 (36.8)	3 (21.4)	3 (30.0)	0.701
Heart disease	1 (5.9)	4 (21.1)	1 (7.1)	0 (0.0)	0.359
Diabetes	3 (17.6)	4 (21.1)	1 (7.1)	0 (0.0)	0.406
Rheumatoid arthritis	1 (5.9)	1 (5.3)	2 (14.3)	0 (0.0)	0.200
Hyperlipidemia	0 (0.0)	4 (21.1)	2 (14.3)	1 (10.0)	0.240
Multiple sclerosis	0 (0.0)	0 (0.0)	0 (0.0)	0 (0.0)	n/a
Psychological disorder	2 (11.8)	4 (21.1)	5 (35.7)	0 (0.0)	0.140
Vision loss	1 (5.9)	4 (21.1)	3 (21.4)	1 (10.0)	0.578
Hearing loss	3 (17.6)	3 (15.8)	4 (28.6)	1 (10.0)	0.746
Sleep apnea	4 (23.5)	4 (21.1)	1 (7.1)	0 (0.0)	0.270
Narcolepsy	0 (0.0)	0 (0.0)	0 (0.0)	0 (0.0)	n/a
Restless leg syndrome	1 (5.9)	4 (21.1)	3 (21.4)	1 (10.0)	0.578

*P-value is reported from the Fisher-Freeman-Halton Exact test
